# Association of caffeine intake with all-cause and cardiovascular mortality in diabetes and prediabetes

**DOI:** 10.1186/s13098-024-01417-6

**Published:** 2024-07-26

**Authors:** Haipeng Yao, Lamei Li, Xiabo Wang, Zhongqun Wang

**Affiliations:** 1 Department of Cardiology, Affiliated Hospital of Jiangsu University, Zhenjiang, China; 2 Institute of Cardiovascular Diseases, Jiangsu University, Zhenjiang, China; 3 Department of Cardiology, Wujin Hospital Affiliated With Jiangsu University, Changzhou, China

**Keywords:** Diabetes, Predibetes, Caffeine, Mortality, NHANES

## Abstract

**Backgroud:**

The association between caffeine intake and mortality in prediabetes and diabetes is not well defined. This study was designed to investigate the association between caffeine intake and all-cause mortality and cardiovascular disease (CVD) mortality in adults with prediabetes and diabetes in the United States.

**Methods:**

This analysis included 18,914 adult patients with diabetes and prediabetes from the National Health and Nutrition Examination Survey (NHANES) 2003–2018. Follow-up extended to December 31, 2019. Weighted Cox proportional hazards regression models were used to estimate the hazard ratios (HR) and 95% confidence intervals (CI) for all-cause mortality and CVD mortality.

**Results:**

During 142,460 person-years of follow-up, there were 3,166 cases of all-cause mortality and 1,031 cases of CVD mortality recorded. In the fully adjusted models, caffeine intake showed a significant dose-response association with the risk of all-cause mortality and CVD mortality in individuals with diabetes and prediabetes. When comparing extreme quartiles of caffeine intake, the multivariable-adjusted hazard ratio for all-cause mortality was 0.78 (0.67–0.91) (*P* for trend = 0.007); however, there was no significant association with the risk of CVD mortality. Results remained consistent in stratified analyses by sex, age, race/ethnicity, education level, family income-poverty ratio, BMI, hypertension, smoking status, alcohol intake, and HEI-2015.

**Conclusions:**

This study suggests that caffeine intake is significantly inversely associated with the risk of all-cause mortality in individuals with diabetes and prediabetes. In individuals with prediabetes, there is also a significant inverse association between caffeine intake and CVD events, but this association is not present in those with diabetes.

**Graphical Abstract:**

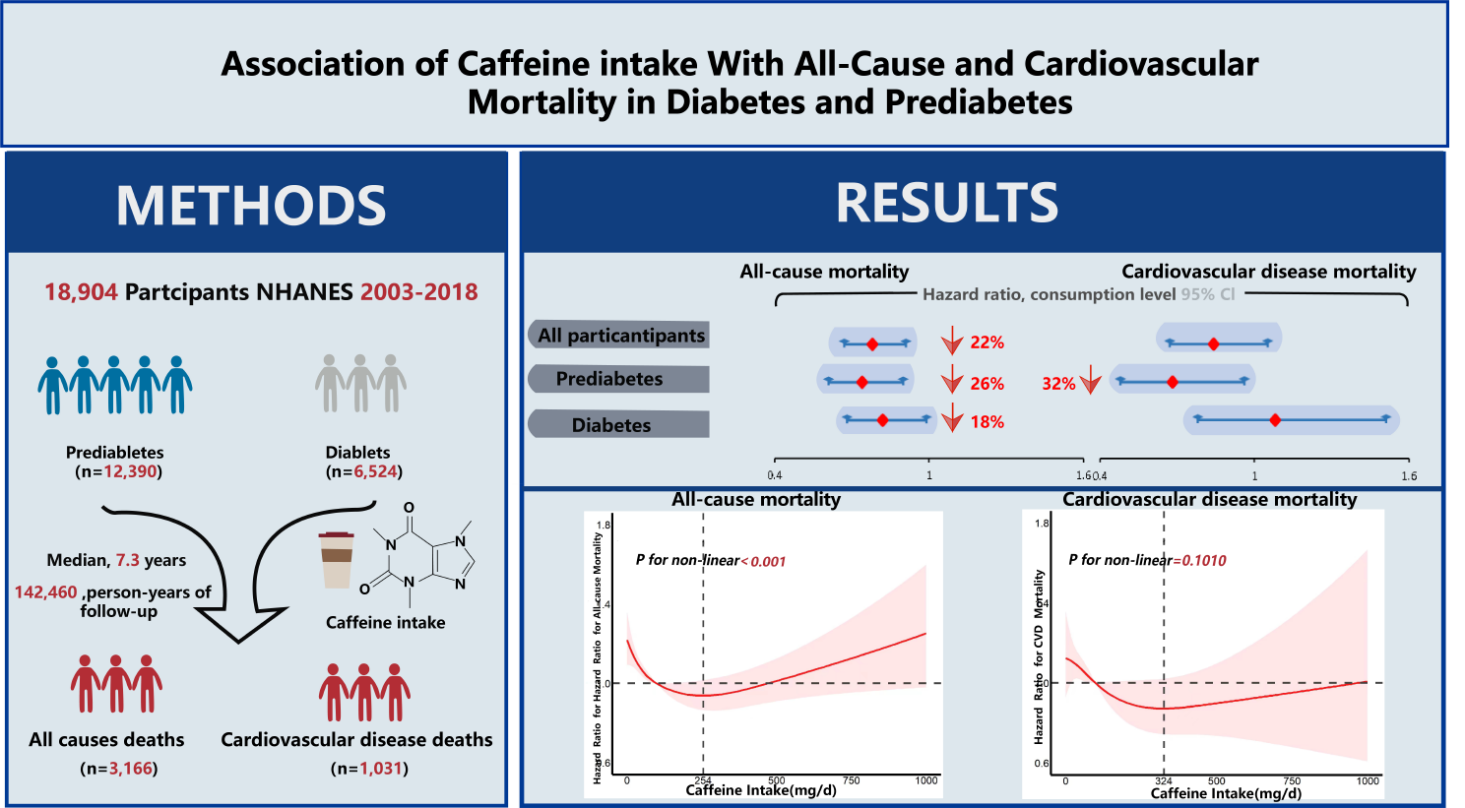

**Supplementary Information:**

The online version contains supplementary material available at 10.1186/s13098-024-01417-6.

## Introduction

Diabetes is a challenging and increasingly prevalent public health issue in the 21st century. In 2021, the global diabetic population had reached 537 million, projected to exceed 700 million by 2045 [1]. Diabetes often develops years before diagnosis, especially when it includes prediabetes [2]. Prediabetes is an intermediate stage between normal blood sugar and diabetes, characterized by impaired glucose metabolism [3]. Diabetes often develops years before diagnosis, especially when it includes prediabetes. Prediabetes is an intermediate stage between normal blood sugar and diabetes, characterized by impaired glucose metabolism. DM and prediabetes are commonly present in patients diagnosed with CVD and are closely associated with poor prognosis [4, 5]. With the diversification of intervention measures, increasing evidence suggests that dietary factors play an important role in preventing adverse outcomes in populations with diabetes and prediabetes [6].

Coffee is one of the most commonly consumed beverages globally. Accumulated evidence shows a negative correlation between coffee consumption and chronic diseases, including CVD [7], diabetes [8], Parkinson’s disease [9], and cancer [10]. In addition, extensive studies on cohorts have also observed a negative correlation between coffee consumption and specific mortality rates, as well as overall mortality risks [11, 12]. Nevertheless, the impact of coffee is still subject to ongoing questioning and concern stemming from the caffeine content in coffee. Although caffeine is considered a rich source of antioxidants and other bioactive compounds, short-term metabolism studies have shown that caffeine can acutely increase blood pressure by antagonizing adenosine A1 and A2A receptors and acutely adversely affect arterial stiffness and endothelium-dependent vasodilation [13, 14]. Cross-sectional studies have shown that coffee consumption may temporarily increase the risk of nonfatal myocardial infarction, ischemic stroke, and sudden death [15]. The effects of coffee have been suggested to be offset by the beneficial influences of other compounds in coffee on the pathogenesis of coronary heart disease. However, recent research has also found a U-shaped relationship between caffeine intake and CVD risk, suggesting that caffeine intake may not increase the risk of CVD within a moderate range.

In the diabetic population, there is limited data on the association between caffeine intake and CVD mortality risk. Although some studies have observed an association with a lower risk of death, most study results suggest no association [16]. However, evidence regarding the cardiovascular effects of caffeine intake in diabetic and prediabetic patients with oxidative stress is scarce.

This study utilized data from the National Health and Nutrition Examination Survey. It aimed to prospectively investigate the association between caffeine intake and the risks of all-cause mortality and CVD mortality among a nationally representative sample of American adults with diabetes and prediabetes.

## Materials and methods

### Study population

The National Health and Nutrition Examination Survey (NHANES) is a continuous, stratified, multistage sampling study managed by the Centers for Disease Control and Prevention (CDC). It aims to assess the health and nutritional status of the U.S. population. The National Center for Health Statistics (NCHS) Institutional Review Board has approved the NHANES research plan, and all participants in the survey have provided written informed consent.

**Fig. 1:**
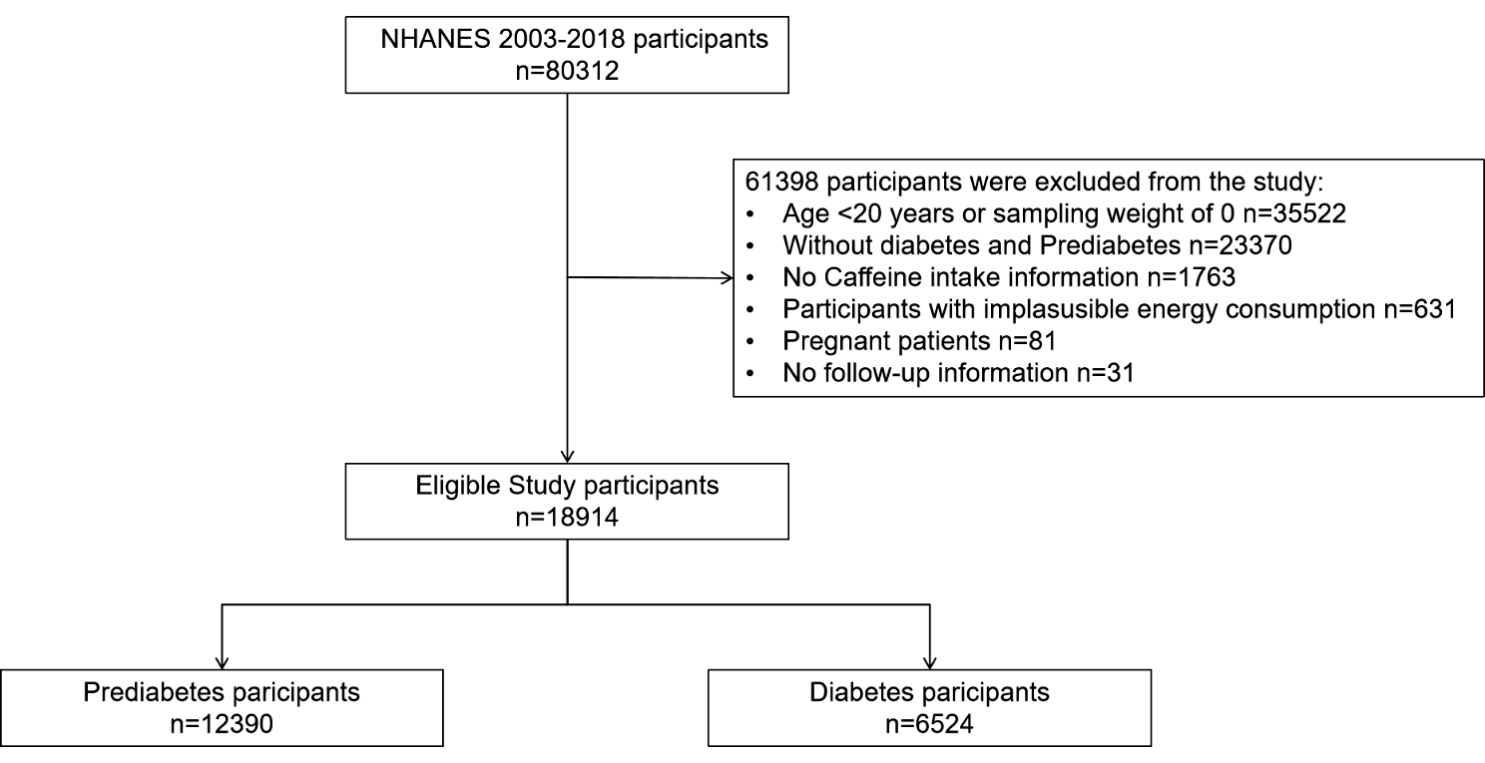
Flowchart of participants in this study

This study included 80,312 participants from NHANES between 2003 and 2018. Exclusion criteria were as follows: (1) participants under 20 years old; (2) participants not defined as having diabetes or prediabetes; (3) participants with missing data on caffeine intake; (4) daily energy intake below 800 kcal or above 4200 kcal for men, and below 500 kcal or above 3500 kcal for women; (5) participants self-reporting pregnancy; (6) lack of follow-up information. Ultimately, 18,914 adult participants with diabetes or prediabetes were included in the final analysis (Fig. 1).

### Assessment of coffeine intake

The U.S. Department of Agriculture (USDA) uses the Automated Multiple-Pass Method to collect 24-hour dietary recall data as part of the “What We Eat In America” component of the NHANES dietary interview. From 2003 to 2018, all NHANES participants were eligible to participate in two 24-hour dietary recall interviews. The first dietary recall interview was conducted at the Mobile Examination Center (MEC), and the second interview was conducted via phone 3 to 10 days later. In this analysis, coffeine intake was assessed using the average of the 2-day food recall interviews, and if only data from the first day is available, that value is used instead of the average.

### Assessment of diabetes and prediabetes

Diabetes is defined as any of the following: self-reported diagnosis of diabetes by a doctor, use of oral hypoglycemic agents or insulin, fasting blood glucose level exceeding 126 mg/dL, or glycated hemoglobin (HbA1c) ≥ 6.5% (*n* = 6524). Prediabetes [17] is identified through self-reported prediabetes status or fasting blood glucose levels between 100 mg/dL and 125 mg/dL or HbA1c between 5.7% and 6.4%, excluding those diagnosed with diabetes (*n* = 12,390).

### Assessment of covariates

To correctly identify potential confounding factors and effect modifiers, we constructed a Directed Acyclic Graph (DAG) [18] to guide the establishment of the regression risk model (Additonal file 1: Fig. S1). Standardized questionnaires were used to collect participants’ socio-demographic characteristics, dietary intake, smoking, duration of diabetes, complications, medication use, hypertension, self-reported heart disease, and malignant tumors. Physical examinations and laboratory tests were conducted by experienced medical personnel in the MEC.

Race/ethnicity is classified as non-Hispanic white, non-Hispanic black, Mexican American, and other races. Educational level is classified as less than high school, high school or equivalent, and college or higher. The Family income-to-poverty ratio is ≤ 1.3, 1.3–3.5, and > 3.5. Smoking status is defined as never smoked (those who reported smoking < 100 cigarettes in their lifetime), former smoker (those who smoked > 100 cigarettes in their lifetime and have quit), and current smoker (those who have smoked > 100 cigarettes in their lifetime).Drinking habit is defined as non-drinker, moderate drinker (0.1–13.9 g/day for women, 0.1–27.9 g/day for men), and heavy drinker (≥ 14 g/day for women, ≥ 28 g/day for men). Body Mass Index (BMI), calculated as weight (in kilograms) divided by height (in meters) squared, was categorized as < 25.0, 25.0-29.9, and ≥ 30. Obesity was defined as BMI ≥ 30 Kg/m^2^. Blood pressure (systolic and diastolic) is determined by taking the average of three qualified measurements. Hypertension is defined as previously diagnosed with hypertension or taking antihypertensive medication or average blood pressure ≥ 140/90 mmHg. The duration of diabetes is classified as ≤ 3 years, 3–10 years, > 10 years.

In addition, the serum concentrations of HDL cholesterol (HDL-C), LDL cholesterol (LDL-C), triglycerides (TG), total cholesterol (TC), and uric acid (SUA) are measured through standardized laboratory testing. The eGFR is calculated using the Chronic Kidney Disease Epidemiology Collaboration (CKD-EPI) equation [19]. Dietary intake and nutrient components, including energy intake, total protein intake, sugar, carbohydrates, dietary fiber, total saturated fatty acids, total polyunsaturated fatty acids, alcohol intake, vitamin E, vitamin A, red meat intake (oz eq), and white meat intake (oz eq) data. According to the 2015–2020 edition of the Dietary Guidelines for Americans (DGA), the 13 dietary components considered in the Health Eating Index(HEI-2015) scoring system indicate overall diet quality. Dietary patterns that emphasize increased consumption of adequate components and decreased intake of moderate components generally correspond to higher HEI-2015 scores (ranging from 0 to 100 points), with higher scores typically indicating better diet quality [20]. Baseline CVD is defined as being diagnosed by a doctor with congestive heart failure, coronary heart disease, heart attack, or stroke. Baseline cancer is identified as being diagnosed by a doctor with cancer or malignancy.

### Assessment of death

The mortality status was determined as of December 31, 2019, through linkage with the national mortality index records. The follow-up period was defined from the baseline to death or the end of follow-up, whichever occurred first. The main causes of death were confirmed based on ICD-10 codes. The codes for cardiovascular disease deaths include I00-I09, I11, I13, I20-I51, and I60-I69.

### Statistical analysis

The NHANES study adopted a multistage, complex, stratified, and sampling design, with all statistical analyses incorporating sample weights, stratification, and primary sampling units to represent the entire population of the United States. Missing values for each variable were calculated first, and variables with missing value proportions exceeding 20% (such as fasting plasma glucose, low-density lipoprotein, and triglycerides) were excluded to ensure the reliability of the results. Multiple imputation methods were used, with five replicates generated using random forest to address the missing data issues in the baseline characteristic variables (Additonal file 1: Fig. S2).

Baseline features were presented as weighted averages ± standard errors or frequencies (percentages). Baseline feature differences among the four groups of caffeine intake levels (Q1-Q4) were compared using the one-way analysis of variance, Kruskal-Wallis H test, or chi-square test. Baseline feature differences between the diabetes and prediabetes groups were compared using t-tests and chi-square tests.

We fitted three models. Model 1 adjusted for sex, age, race/ethnicity (non-Hispanic white, non-Hispanic black, Mexican American, and other races), BMI (< 25.0, 25.0-29.9, ≥ 30), education level (less than high school, high school or equivalent, university or higher), family income-to-poverty ratio (≤ 1.3, 1.3–3.5, > 3.5), smoking status (never smoked, former smoker, current smoker), alcohol intake (non-drinker, moderate, heavy), and hypertension. Model 2 was further adjusted for eGFR (continuous), HbA1c (< 7%, ≥ 7%), HDL-C (continuous), TC (continuous), and SUA (continuous). Model 3 was further adjusted for HEI-2015 (continuou), red meat intake (oz. eq./d, continuous), white meat intake (oz. eq./d, continuous), total protein (continuous), carbohydrate intake (continuous), total sugars (continuous), total energy (continuous), total saturated fatty acids (continuous), total polyunsaturated fatty acids (continuous), dietary fiber (continuous), vitamin E (continuous), vitamin A (continuous). We used the first quartile (Q1) of caffeine intake as the reference group and calculated linear trends by assigning the median value for each category as a continuous variable.

To investigate the dose-response relationship between caffeine intake and all-cause mortality, as well as CVD mortality, we further applied a restricted cubic spline (RCS) regression model without considering weights. Four nodes were set at the 5th, 35th, 65th, and 95th percentiles of caffeine intake distribution. To determine whether the optimal model is linear or nonlinear, we conducted a likelihood ratio test and calculated the p-value for nonlinearity.

Stratified analyses were conducted based on gender, age, race/ethnicity, educational level, family income-to-poverty ratio, smoking status, alcohol intake, hypertension, BMI and HEI-2015. Multiplicative interaction terms between caffeine intake and stratification variables were tested to examine potential effect modification.

Multiple sensitivity analyses were performed to test the robustness of the study results. First, considering the reliability of using data with multiple imputations, we removed participants with missing variablesf(*n* = 4441). Second, to minimize the potential for reverse causality, participants who died within two years after the baseline examination were excluded (*n* = 1131). Third, CVD and cancer are closely related to overall mortality and cardiovascular mortality at baseline. In order to eliminate their confounding effects, participants with CVD and cancer at baseline were excluded (*n* = 4845). Fourth, to examine the impact of diabetes medication use on mortality outcomes, participants using diabetes medications (such as glucose-lowering drugs or insulin) were excluded (*n* = 3711). Fifth, subgroup analyses were conducted for participants with diabetes to examine the relationship between caffeine intake and mortality outcomes and an RCS regression model was established to demonstrate the dose-response association between caffeine intake and mortality outcomes in this diabetes population (*n* = 6524). Sixth, similarly, an analysis was conducted on participants with prediabetes (*n* = 12,390). Seventh, considering some indicators may affect the associations, further adjustments were made for fasting plasma glucose, LDL-C, and TG in the non-multiple imputation models. Lastly, we considered the heterogeneity in participant demographics, dietary intake, and laboratory indicators by incorporating the lowest and extreme quartiles with propensity score matching(PSM) without replacement. This was done to adjust for potential confounders when matching participants with baseline caffeine intake in Q1 to Q4. We utilized a logistic regression model to obtain propensity scores and used a caliper value of 0.1 standard deviations of the logit of the propensity score as the threshold. After matching, 5018 participants were included in the study.

As additional analysis, we will convert caffeine intake into the number of cups consumed (with each cup of coffee containing 100 milligrams of caffeine) to assess the association between coffee consumption and mortality rates.

All analyses were performed using *R* software (version 4.3.1). Two-sided *P*-values < 0.05 were considered statistically significant.

## Results

### Baseline characteristics of participants

The study included 18,914 participants aged 20 and above with diabetes or prediabetes (average age 54.8 years; 51.0% male). Table 1. summarizes the baseline characteristics of participants based on quartiles of caffeine intake. Participants with higher caffeine intake were older, more likely to be female, non-Hispanic white, have higher family income, higher education level, more likely to be current smokers and heavy alcohol drinkers. In addition, these participants seemed to be more likely to be obese and have lower HEI-2015 scores. Those with higher caffeine intake had higher levels of SUA and HDL-C and lower eGFR levels. Additionally, they were more likely to consume higher amounts of red meat, protein, sugar, carbohydrates, energy, dietary fiber, vitamin E, and vitamin A. Similar results were obtained in the population without multiple imputations (Additonal file 1: Table S1). Approximately 20.6%, 44.8%, and 14.0% of diabetic participants used insulin, oral hypoglycemic agents, and had diabetic retinopathy, respectively. Compared to diabetic participants, those with prediabetes had higher caffeine intake (Additonal file 1: Table S2).

**Table 1:** Baseline characteristics of participants with quartiles of caffeine intake among diabetes and prediabetes participants

Characteristic	Caffeine intake(mg/d)					*P*
	Total	Quartile 1 ≤ 29.0	Quartile 2 29.1–98.0	Quartile 3 98.1–200.0	Quartile 4 > 200.0	
Patients, n	18,914	4772	4723	4699	4720	
Age, years	54.8 ± 15.7	54.2 ± 17.6	53.5 ± 16.6	55.1 ± 15.8	56.0 ± 13.4	< 0.001
Sex, n(%)						< 0.001
Male	9,746 (51.0%)	2,219 (45.7%)	2,207 (44.5%)	2,419 (48.7%)	2,901(60.5%)	
Female	9,168 (49.0%)	2,553 (54.3%)	2,516 (55.5%)	2,280 (51.3%)	1,819 (39.5%)	
Race/ethnicity, n(%)						< 0.001
Non-Hispanic White	7,652 (64.8%)	1,351 (49.9%)	1,378 (52.3%)	1,974 (66.6%)	2,949 (81.5%)	
Non-Hispanic Black	4,401 (12.9%)	1,689 (24.3%)	1,302 (17.4%)	915 (10.4%)	495 (4.4%)	
Mexican American	3,203 (8.8%)	838 (11.1%)	969 (12.9%)	823 (8.7%)	573 (4.7%)	
Other	3,658 (13.5%)	894 (14.8%)	1,074 (17.5%)	987 (14.4%)	703 (9.4%)	
Education level, n(%)						< 0.001
Less than high	5,489 (19.1%)	1,575 (23.5%)	1,552 (22.6%)	1,304 (18.1%)	1,058 (14.8%)	
High school grad or equivalent	4,488 (25.4%)	1,104 (24.0%)	1,095 (26.1%)	1,124 (26.4%)	1,165 (25.2%)	
College or above	8,937 (55.4%)	2,093 (52.5%)	2,076 (51.3%)	2,271 (55.6%)	2,497 (60.0%)	
Family income-poverty ratio, n(%)						< 0.001
≤ 1.30	6,045 (22.8%)	1,749 (29.6%)	1,652 (27.4%)	1,417 (21.7%)	1,227 (16.3%)	
1.3–3.5	7,426 (37.3%)	1,881 (39.2%)	1,863 (39.1%)	1,884 (37.1%)	1,798 (34.9%)	
> 3.5	5,443 (39.9%)	1,142 (31.3%)	1,208 (33.5%)	1,398 (41.3%)	1,695 (48.7%)	
BMI, Kg/m2						< 0.001
< 25.0	3,638 (18.8%)	968 (20.5%)	930 (19.9%)	894 (19.1%)	846 (16.9%)	
25.0-29.9	6,138 (31.6%)	1,436 (28.4%)	1,515 (29.3%)	1,585 (32.1%)	1,602 (35.0%)	
≥ 30	9,138 (49.5%)	2,368 (51.1%)	2,278 (50.8%)	2,220 (48.8%)	2,272 (48.1%)	
Smoking status, n(%)						< 0.001
Never	9,749 (50.7%)	2,964 (63.0%)	2,793 (60.3%)	2,355 (50.0%)	1,637 (36.8%)	
Former smoker	5,592 (30.1%)	1,201 (24.4%)	1,251 (25.0%)	1,474 (32.9%)	1,666 (35.1%)	
Current smoker	3,573 (19.2%)	607 (12.6%)	679 (14.7%)	870 (17.1%)	1,417 (28.1%)	
Alcohol intake, n(%)						< 0.001
Never	14,335 (72.4%)	3,769 (76.8%)	3,683 (75.5%)	3,526 (71.5%)	3,357 (68.2%)	
Moderate	2,666 (15.6%)	556 (12.6%)	636 (14.4%)	706 (16.7%)	768 (17.6%)	
Heavy	1,913 (12.0%)	447 (10.6%)	405 (10.1%)	467 (11.9%)	594 (14.2%)	
Hypertension, n(%)						0.200
No	8,223 (46.9%)	1,939 (44.5%)	2,100 (47.5%)	2,080 (47.9%)	2,104 (47.2%)	
Yes	10,691 (53.1%)	2,833 (55.5%)	2,623 (52.5%)	2,619 (52.1%)	2,616 (52.8%)	
Insulin therapy, n(%)						0.700
No	17,550 (93.9%)	4,395 (93.4%)	4,402 (94.0%)	4,358 (93.8%)	4,395 (94.2%)	
Yes	1,364 (6.1%)	377 (6.6%)	321 (6.0%)	341 (6.2%)	325 (5.8%)	
Oral hypoglycemic drugs, n(%)						0.500
No	15,992 (86.8%)	4,053 (87.7%)	3,977 (86.7%)	3,934 (86.7%)	4,028 (86.2%)	
Yes	2,922 (13.2%)	719 (12.3%)	746 (13.3%)	765 (13.3%)	692 (13.8%)	
Diabetic retinopathy, n(%)						0.043
No	17,890 (95.9%)	4,494 (95.4%)	4,441 (95.3%)	4,474 (96.6%)	4,481 (96.0%)	
Yes	1,024 (4.1%)	278 (4.6%)	282 (4.7%)	225 (3.4%)	239 (4.0%)	
Diabetes	6,524 (29.5%)	1,724 (30.8%)	1,657 (30.5%)	1,643 (29.9%)	1,500 (27.9%)	0.110
Prediabetes	12,390 (70.5%)	3,048 (69.2%)	3,066 (69.5%)	3,056 (70.1%)	3,220 (72.1%)	0.110
Diabetes duration						0.300
≤3 years	14,139 (79.0%)	3,528 (78.8%)	3,526 (78.5%)	3,465 (77.9%)	3,620 (80.4%)	
3–10 years	2,106 (9.7%)	551 (9.8%)	537 (10.0%)	520 (9.8%)	498 (9.4%)	
>10 years	2,669 (11.2%)	693 (11.4%)	660 (11.5%)	714 (12.3%)	602 (10.1%)	
HbA_1c_(%)						0.004
<7	16,001 (87.6%)	4,046 (88.3%)	3,944 (85.6%)	3,941 (87.3%)	4,070 (88.7%)	
≥7	2,913 (12.4%)	726 (11.7%)	779 (14.4%)	758 (12.7%)	650 (11.3%)	
eGFR(mL/min/1.73m^2^)	89.6 ± 21.7	89.1 ± 23.8	90.6 ± 23.3	89.6 ± 21.6	89.3 ± 18.9	0.022
Uric acid(mg/dL)	5.7 ± 1.4	5.6 ± 1.5	5.6 ± 1.4	5.7 ± 1.4	5.8 ± 1.4	0.001
HDL-C (mg/dL)	50.9 ± 15.5	51.6 ± 16.0	50.5 ± 15.1	51.6 ± 16.1	50.3 ± 14.8	0.006
TC(mg/dL)	197.1 ± 43.8	192.9 ± 43.2	197.5 ± 43.3	198.4 ± 45.5	198.6 ± 43.1	< 0.001
HEI-2015	49.9 ± 11.9	52.0 ± 12.4	49.9 ± 11.7	49.7 ± 12.1	48.7 ± 11.4	< 0.001
Total energy(Kcal/d)	1,912.0 (1,481.0, 2,442.7)	1,750.8 (1,342.9, 2,239.5)	1,817.0 (1,394.0, 2,319.4)	1,906.6 (1,489.0, 2,415.4)	2,079.5 (1,646.1, 2,652.0)	< 0.001
Total carbohydrate intake(g/d)	224.7 (170.5, 292.1)	212.1 (159.0, 270.1)	220.5 (166.0, 282.2)	222.3 (171.5, 295.3)	236.6 (183.9, 306.5)	< 0.001
Total protein(g/d)	79.6 ± 31.6	75.5 ± 31.5	75.8 ± 30.1	78.5 ± 30.2	85.7 ± 32.8	< 0.001
Total sugars (g/d)	92.0 (62.2, 133.3)	84.2 (56.3, 121.2)	91.5 (62.4, 128.3)	91.4 (62.6, 137.3)	96.9 (64.9, 141.9)	< 0.001
Dietary fiber(g/d)	15.1 (10.7, 20.8)	14.9 (10.5, 20.9)	14.3 (10.1, 20.3)	15.2 (10.7, 20.5)	15.7 (11.1, 21.4)	< 0.001
Total saturated fatty acids (g/d)	23.6 (16.4, 32.8)	20.5 (13.8, 29.5)	21.5 (15.1, 29.9)	23.6 (16.9, 32.4)	27.2 (19.4, 37.2)	< 0.001
Total polyunsaturated fatty acids (g/d)	16.1 (11.0, 22.7)	14.6 (9.7, 20.6)	15.1 (10.2, 21.3)	16.1 (11.2, 23.0)	17.9 (12.6, 24.7)	< 0.001
Vitamin E(mg/d)	6.9 (4.8, 9.9)	6.4 (4.4, 9.2)	6.5 (4.4, 9.3)	6.9 (4.9, 9.7)	7.7 (5.3, 10.7)	< 0.001
Vitamin A(mg/d)	0.5 (0.3–0.8)	0.5(0.3–0.8)	0.5(0.3–0.8)	0.5(0.3–0.8)	0.6(0.4–0.8)	< 0.001
Red meat intake(oz. eq./d)	1.1 (0.0, 2.5)	0.8 (0.0, 2.3)	1.1 (0.0, 2.3)	1.2 (0.0, 2.5)	1.3 (0.0, 2.8)	< 0.001
White meat intake(oz. eq./d)	0.8 (0.0, 2.3)	0.9 (0.0, 2.4)	0.8 (0.0, 2.3)	0.8 (0.0, 2.2)	0.8 (0.0, 2.2)	0.400

Continuous variables with a normal distribution are described as the weighted averages ± standard errors (SE), while continuous variables with a non-normal distribution are described as the median (interquartile range). Categorical variables are represented by numbers (weighted percentages). BMI: body mass index. HDL-C: HDL cholesterol. TC: total cholesterol. HEI: Health eating index

### Association of caffeine intake with mortality in patients with diabetes and prediabetes

**Fig. 2:**
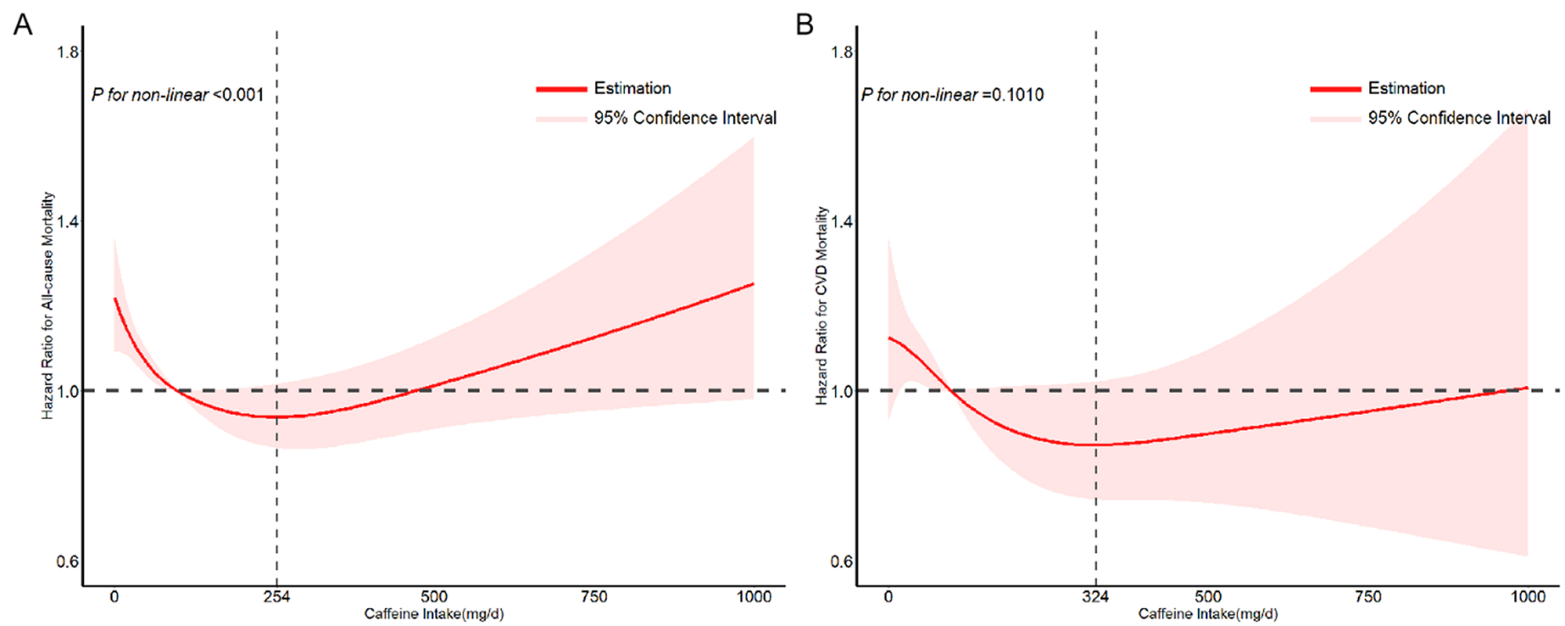
Multivariable adjusted restricted cubic splines forassociations of caffeine intake with all-cause and cardiovascular mortality among diabetes and prediabetes from NHANES 2003–2018. **A**: Dose-response relationship between caffeine intake and all-cause mortality among diabetes and prediabetes in NHANES 2003–2018. P for non linear < 0.001. **B**: Dose-response relationship between caffeine intake and cardiovascular mortality among diabetes and prediabetes in NHANES 2003–2018. P for non linear = 0.1010. The four nodes of the restricted cubic spline (RCS) are set at the 5th, 35th, 65th, and 95th percentiles of caffeine intake distribution. To determine whether the optimal model is linear or non-linear, we conducted a likelihood ratio test and calculated the p-value for non-linearity. Complete model were adjusted for sex (female or male), age (continuous), race/ethnicity (non-Hispanic white, non-Hispanic black, Mexican American, and other races), BMI (< 25.0, 25.0-29.9, ≥ 30), education level (less than high school, high school or equivalent, university or higher), family income-to-poverty ratio (≤ 1.3, 1.3–3.5, > 3.5), smoking status (never smoked, former smoker, current smoker), alcohol intake (non-drinker, moderate drinker, heavy drinker), hypertension (yes or no), eGFR (continuous), HbA1c (< 7%, ≥ 7%), HDL-C (continuous), TC (continuous), SUA (continuous), HEI-2015 (continuous), red meat intake (oz. eq./d, continuous), white meat intake (oz. eq./d, continuous), total protein (continuous), carbohydrate intake (continuous), total sugars (continuous), total energy (continuous), total saturated fatty acids (continuous), total polyunsaturated fatty acids (continuous), dietary fiber (continuous), vitamin E (continuous), vitamin A (continuous). The pale pink shaded area represents a 95% confidence interval

During the 142,460-person-years of follow-up (with an average follow-up time of 7.3 years), a total of 3166 deaths were recorded, including 1031 deaths from CVD. After multivariable adjustment, higher caffeine intake was significantly associated with reduced risks of all-cause mortality in participants with diabetes and prediabetes. In the fully adjusted model, multivariable-adjusted hazard ratios (HRs) and 95% confidence intervals (CIs) for quartiles of caffeine intake were as follows: Q1, 1.00 (reference); Q2, 0.85 (0.74–0.97); Q3, 0.78 (0.68–0.90); and Q4, 0.78 (0.67–0.91) (*P* for trend = 0.007). No significant association was observed between quartiles of caffeine intake and CVD mortality rates (Table 2). Further validation revealed a linear dose-response relationship between caffeine intake (ranging from 0 to 1000 mg/day) and all-cause mortality (*P* for non-linear < 0.001) as well as CVD mortality (*P* for non-linear = 0.101), exhibiting a nearly U-shaped curve (Fig. 2).

**Table 2:** Multivariate adjustment analysis of caffeine intake and all-cause and cardiovascular mortality in patients with diabetes and prediabetes in NHANES 2003–2018

Categories	Quartiles of caffeine intake (mg/d)				*P* for trend
	Quartile 1 ≤ 29.0	Quartile 2 29.1–98.0	Quartile 3 98.1–200.0	Quartile 4 > 200.0	
**All-cause mortality**					
No. deaths/total	879/4772	748/4723	746/4699	793/4720	
Crude	1	0.76(0.67–0.86)	0.75(0.65–0.87)	0.75(0.65–0.86)	0.004
Model1	1	0.87(0.75–1.01)	0.81(0.7–0.93)	0.79(0.68–0.93)	0.007
Model2	1	0.86(0.75-1)	0.80(0.69–0.91)	0.79(0.68–0.93)	0.008
Model3	1	0.85(0.74–0.97)	0.78(0.68–0.9)	0.78(0.67–0.91)	0.007
**CVD mortality**					
No. deaths/total	290/4772	260/4723	240/4699	241/4720	
Crude	1	0.80(0.64-1)	0.78(0.62–0.99)	0.7(0.56–0.86)	0.006
Model1	1	0.94(0.74–1.19)	0.89(0.71–1.12)	0.84(0.67–1.06)	0.140
Model2	1	0.93(0.73–1.18)	0.88(0.7–1.1)	0.84(0.68–1.06)	0.146
Model3	1	0.91(0.72–1.15)	0.86(0.69–1.08)	0.84(0.67–1.05)	0.147

Data are presented as HR (95% CI)

Model 1: adjusted for sex (female or male), age (continuous), race/ethnicity (non-Hispanic white, non-Hispanic black, Mexican American, and other races), BMI (< 25.0, 25.0-29.9, ≥ 30), education level (less than high school, high school or equivalent, university or higher), family income-to-poverty ratio (≤ 1.3, 1.3–3.5, > 3.5), smoking status (never smoked, former smoker, current smoker), alcohol intake (non-drinker, moderate drinker, heavy drinker), and hypertension (yes or no)

Model 2: Model 1 + eGFR (continuous), HbA1c (< 7%, ≥ 7%), HDL-C (continuous), TC (continuous), and SUA (continuous)

Model 3: Model 2 + HEI-2015 (continuou), red meat intake (oz. eq./d, continuous), white meat intake (oz. eq./d, continuous), total protein (continuous), carbohydrate intake (continuous), total sugars (continuous), total energy (continuous), total saturated fatty acids (continuous), total polyunsaturated fatty acids (continuous), dietary fiber (continuous), vitamin E (continuous), vitamin A (continuous)

### Subgroup analysis

According to gender (female and male), age (≤ 60 and > 60), race/ethnicity (non-Hispanic white, other races), education level (high school or equivalent and below, college and above), family income-to-poverty ratio (≤ 1.3, 1.3–3.5, > 3.5), smoking status (non-smoker, former smoker, current smoker), alcohol consumption (never drinker, moderate drinker, heavy drinker), hypertension (yes or no), BMI (< 30, ≥ 30 kg/m2), and HEI-2015(in quartiles), stratified analysis was conducted (Table 3 and Table 4). The results showed a significant interaction between age groups and smoking status with all-cause mortality within each subgroup, while no significant interaction was found in other subgroups.

**Table 3:** Stratified analysis of the association between caffeine intake and all-cause mortality in patients with diabetes and prediabetes in NHANES 2003–2018

Characteristics	No. deaths/total	Caffeine intake (mg/d)				*P* for trend	*P* interaction
		Quartile 1	Quartile 2	Quartile 3	Quartile 4		
Sex							0.217
Male	1808/9746	1	0.90(0.75–1.08)	0.70(0.59–0.85)	0.85(0.7–1.05)	0.239	
Female	1358/9168	1	0.79(0.66–0.96)	0.81(0.67–0.98)	0.72(0.58–0.9)	0.014	
Age, years							0.007
≤ 60	543/10,287	1	0.71(0.51–0.99)	0.76(0.52–1.13)	0.87(0.6–1.27)	0.995	
> 60	2623 /8627	1	0.85(0.73–0.99)	0.76(0.66–0.88)	0.74(0.62–0.87)	0.001	
Race/ethnicity							0.758
Non-Hispanic White	1860/7610	1	0.92(0.81–1.07)	0.76(0.65–0.89)	0.84(0.69–1.04)	0.057	
Other	1289/11,262	1	0.96(0.7–1.32)	0.85(0.63–1.17)	0.85(0.57–1.29)	0.168	
Education level							0.749
Less college	2035/9977	1	0.83(0.71–0.98)	0.82(0.69–0.99)	0.90(0.75–1.09)	0.655	
College or high	1131/8937	1	0.86(0.67–1.11)	0.61(0.49–0.77)	0.70(0.54–0.91)	0.008	
Family income-poverty ratio							0.253
1.3	1153/6045	1	0.88(0.70–1.11)	0.90(0.71–1.14)	0.82(0.65–1.04)	0.153	
1.3–3.5	1393/7426	1	0.73(0.60–0.89)	0.80(0.67–0.95)	0.73(0.60–0.90)	0.023	
> 3.5	192/5443	1	0.98(0.73–1.33)	0.72(0.53–0.99)	0.87(0.60–1.26)	0.389	
Hypetension							0.143
No	786/8223	1	0.89(0.66–1.20)	0.85(0.64–1.12)	0.86(0.64–1.14)	0.393	
Yes	2308/10,691	1	0.80(0.68–0.93)	0.75(0.65–0.86)	0.76(0.65–0.89)	0.007	
BMI, kg/m^2^							0.391
< 30	1847/9906	1	0.82(0.70–0.98)	0.78(0.67–0.92)	0.78(0.64–0.94)	0.032	
≥ 30	1319/9008	1	0.82(0.65–1.03)	0.75(0.59–0.94)	0.75(0.60–0.95)	0.051	
Smoking status							0.022
Never	1247/9749	1	0.82(0.66–1.01)	0.86(0.70–1.06)	0.78(0.62–0.99)	0.108	
former smoker	1344/5592	1	0.76(0.63–0.91)	0.78(0.64–0.95)	0.84(0.71–0.99)	0.147	
Current smoker	575/3573	1	0.91(0.62–1.34)	0.68(0.45–1.03)	0.79(0.55–1.15)	0.262	
Alcohol intake							0.518
Never	2555/14,335	1	0.86(0.75–0.99)	0.85(0.73–0.99)	0.85(0.74-1.00)	0.175	
Moder	377/2662	1	0.66(0.42–1.02)	0.61(0.40–0.96)	0.72(0.48–1.10)	0.272	
Heavy	232/1902	1	0.70(0.43–1.14)	0.49(0.31–0.77)	0.53(0.30–0.95)	0.058	
HEI-2015							0.144
Q1 ≤ 41.7	747/4721	1	0.75(0.56-1)	0.79(0.57–1.1)	0.88(0.65–1.2)	0.827	
Q2 41.8–49.7	831/4756	1	0.91(0.68–1.2)	0.74(0.56–0.98)	0.84(0.62–1.15)	0.345	
Q3 49.8–58.4	806/4721	1	0.65(0.49–0.87)	0.79(0.6–1.04)	0.73(0.54–0.97)	0.185	
Q4 > 58.4	782/4716	1	0.77(0.6–0.98)	0.82(0.66–1.03)	0.65(0.49–0.85)	0.004	

Data are presented as HR (95% CI). Adjusted for sex, age, race/ethnicity, BMI, education level, family income-to-poverty ratio, smoking status, alcohol intake, hypertension, eGFR, HbA1c, HDL-C, TC, SUA, HEI-2015, red meat intake, white meat intake, total protein, carbohydrate intake, total sugars, total energy, total saturated fatty acids, total polyunsaturated fatty acids, dietary fiber, vitamin E, vitamin A.The strata variable was not included in the model when stratifying by itself

**Table 4:** Stratified analysis of the association between caffeine intake and cardiovascular mortality in patients with diabetes and prediabetes in NHANES 2003–2018

Characteristics	No. deaths/total	Caffeine intake (mg/d)				*P* for trend	*P* interaction
		Quartile 1	Quartile 2	Quartile 3	Quartile 4		
Sex							0.833
Male	595/9746	1	1.31(1.00-1.72)	0.87(0.63–1.22)	0.87(0.62–1.24)	0.148	
Female	436/9168	1	0.73(0.51–1.05)	0.70(0.51–0.97)	0.93(0.67–1.31)	0.92	
Age, years							0.55
≤ 60	143/10,287	1	1.11(0.62-2)	1.27(0.72–2.24)	1.32(0.66–2.63)	0.469	
> 60	888 /8627	1	0.89(0.7–1.12)	0.77(0.61–0.97)	0.73(0.58–0.93)	0.011	
Race/ethnicity							0.431
Non-Hispanic White	605/7610	1	0.95(0.76–1.21)	0.89(0.68–1.17)	0.98(0.71–1.37)	0.852	
Other	419/11,262	1	0.86(0.75–0.99)	0.85(0.73–0.99)	0.85(0.74-1.00)	0.432	
Education level							0.205
Less college	663/9977	1	0.91(0.72–1.15)	0.90(0.67–1.23)	1.18(0.88–1.59)	0.158	
College or high	368/8937	1	0.91(0.64–1.30)	0.61(0.43–0.89)	0.70(0.48–1.02)	0.062	
Family income-poverty ratio							0.099
1.3	372/6045	1	0.95(0.64–1.42)	0.92(0.61–1.38)	1.00(0.71–1.41)	0.911	
1.3–3.5	467/7426	1	0.74(0.53–1.05)	0.69(0.51–0.93)	0.74(0.54–1.01)	0.133	
> 3.5	620/5443	1	1.85(1.16–2.95)	1.38(0.78–2.46)	1.14(0.56–2.35)	0.789	
Hypetension							0.596
No	218/8223	1	1.19(0.72-1.97)	0.9(0.46-1.77)	0.87(0.48-1.58)	0.45	
Yes	813/10,691	1	0.85(0.68-1.08)	0.84(0.66-1.07)	0.86(0.69-1.09)	0.373	
BMI, kg/m^2^							0.532
< 30	571/9906	1	0.90(0.69–1.16)	0.80(0.59–1.08)	0.82(0.60–1.13)	0.271	
≥ 30	460/9008	1	0.90(0.63–1.30)	0.83(0.62–1.13)	0.77(0.56–1.07)	0.153	
Smoking status							0.104
Never	456/9749	1	0.85(0.6–1.21)	0.74(0.52–1.06)	0.74(0.50–1.10)	0.173	
former smoker	409/5592	1	0.97(0.68–1.37)	1.01(0.71–1.44)	0.92(0.66–1.27)	0.646	
Current smoker	166/3573	1	1.22(0.65–2.32)	0.90(0.49–1.69)	1.11(0.60–2.08)	0.856	
Alcohol intake							0.319
Never	831/14,335	1	0.84(0.67–1.07)	0.91(0.71–1.17)	0.91(0.71–1.16)	0.711	
Moder	138/2662	1	0.96(0.46-2.00)	0.57(0.28–1.14)	0.74(0.36–1.50)	0.327	
Heavy	62/1902	1	1.67(0.76–3.7)	0.67(0.25–1.78)	0.97(0.30–3.17)	0.618	
HEI-2015							0.073
Q1 ≤ 41.7	241/4721	1	0.77(0.48–1.23)	0.72(0.43–1.21)	0.87(0.56–1.37)	0.82	
Q2 41.8–49.7	278/4756	1	1.09(0.66–1.8)	0.78(0.51–1.2)	0.93(0.6–1.91)	0.858	
Q3 49.8–58.4	260/4721	1	0.77(0.49–1.2)	0.98(0.66–1.48)	0.77(0.49–1.2)	0.375	
Q4 > 58.4	252/4716	1	0.8(0.54–1.19)	1.04(0.67–1.63)	0.61(0.38–0.98)	0.061	

Data are presented as HR (95% CI). Adjusted for sex, age, race/ethnicity, BMI, education level, family income-to-poverty ratio, smoking status, alcohol intake, hypertension, eGFR, HbA1c, HDL-C, TC, SUA, HEI-2015, red meat intake, white meat intake, total protein, carbohydrate intake, total sugars, total energy, total saturated fatty acids, total polyunsaturated fatty acids, dietary fiber, vitamin E, vitamin A.The strata variable was not included in the model when stratifying by itself

### Sensitivity analysis

In sensitivity analysis, firstly, we did not perform multiple imputations for missing values, and the relationship between caffeine intake and overall mortality and CVD mortality did not change significantly (Additonal file 1: Table S3). Additionally, when excluding participants who died within the first two years of follow-up, the negative correlation between caffeine intake and overall mortality rate and CVD mortality rate did not show significant changes (Additonal file 1: Table S4). Furthermore, similar results were observed when participants who self-reported cardiovascular diseases and cancer at baseline were excluded (Additonal file 1: Table S5). Sensitivity analysis, excluding participants who were using diabetes medications (oral hypoglycemic drugs or insulin), yielded similar results for overall mortality rate and CVD mortality rate (Additonal file 1: Table S6). Furthermore, we separately analyzed participants with diabetes and pre-diabetes, and interestingly, this association seemed to be more pronounced in the pre-diabetic population (Additonal file 1: Table S7 and Table S7). RCS analysis also confirmed a near U-shaped association between caffeine intake and mortality in both diabetes and pre-diabetes populations(Additonal file 1: Fig. S3 and Fig. S4). The beneficial effects of caffeine intake at the extreme quartile were hardly affected even after further adjustment for plasma fasting blood glucose, LDL-C, and TG (Additonal file 1: Table S9). After propensity score matching, both groups (Q1: 2509 participants and Q4: 2509 participants) were well balanced across all baseline covariates (standardized mean differences < 0.1) (Additonal file 1: Table S10 and Fig. S5), and the observed risk associations between extreme caffeine intake and overall mortality rate and CVD mortality rate were similar to those obtained before matching (Additonal file 1: Table S11).

### Additional analysis

Lastly, as a supplementary analysis, daily coffee consumption was negatively correlated with all-cause mortality rates but not associated with CVD mortality rates (Additonal file 1: Table S12).

## Disscusion

In the prospective cohort study of American adults with diabetes and prediabetes, we observed a non-linear negative correlation between caffeine intake and the risk of all-cause mortality, showing an approximate U-shaped curve. The association between caffeine intake and CVD mortality was only detected in the prediabetes population. This association was adjusted for traditional confounding factors such as demographic characteristics, lifestyle and socioeconomic factors, cardiovascular risk factors, diet, and medication use. Stratified analysis and sensitivity analysis demonstrated the robustness of our research findings. In the United States, 85% of adults consume caffeine daily, with an average intake of 135 milligrams per day, equivalent to about 1.5 standard cups of coffee. A result from a prospective cohort study of 36 studies on coffee consumption and CVD risk, involving 1,279,804 participants and 36,352 cases of CVD, suggests that moderate coffee consumption (3–5 cups per day) is associated with lower CVD risk [7]. Another queue study shows a clear negative correlation between overall mortality and cardiovascular mortality. Compared to not drinking coffee, consuming ≥ 4 cups of coffee (including caffeine) per day reduces the risks of overall mortality and cardiovascular mortality by 20% and 21% respectively [21]. On the contrary, several other research findings suggest that high coffee intake is positively associated with overall mortality and risk of CVD mortality [22, 23]. In fact, in the 2021 European Society of Cardiology guidelines, it is described that consuming 3–4 cups of coffee per day is moderately beneficial in preventing CVD [24], although such a recommendation was not made in the 2019 American Heart Association/American College of Cardiology guidelines.

In the general population, there may be a U-shaped or J-shaped relationship between coffee consumption and health outcomes [7], suggesting adverse effects of excessive coffee intake. High doses of caffeine intake may lead to various adverse effects, such as increased sympathetic nervous system activity, resulting in autonomic imbalance [25]. For patients with CVD, autonomic imbalance may lead to poor prognosis. Due to variations in metabolism and sensitivity to caffeine among different individuals, it may be necessary to adjust the intake levels accordingly. It is recommended that adults consume no more than 400 milligrams of caffeine per day, while pregnant or lactating women should limit their daily intake to no more than 200 milligrams. There is currently no consensus on the appropriate target for daily caffeine intake for diabetes and prediabetes patients. The risk of all-cause mortality in diabetes patients shows a negative correlation. For example, a cohort study involving 15,486 type 2 diabetes patients found that coffee intake was negatively correlated with all-cause mortality, with a non-linear decrease in risk [26]. People carefully analyze the independent intake of coffee with or without caffeine, as short-term experiments have shown that caffeine intake can significantly increase blood pressure [13], homocysteine levels, and postprandial blood sugar levels [27]. However, results from prospective cohort studies indicate that long-term coffee consumption is not significantly associated with the risk of hypertension. The short-term detrimental effects of caffeine intake on blood pressure can be reduced through partial tolerance developed with long-term use and counteracting effects of other coffee components. It is worth noting that due to the complex physiological effects of coffee and because long-term use typically results in at least partial tolerance to the hemodynamic effects of caffeine, it is difficult to infer the effects of short-term metabolism studies on coffee from long-term use. In another cohort study of 3,948 diabetes patients, an association between caffeine intake and overall mortality risk was only found in female patients [28]. However, a cohort study in Scotland found [29] that higher coffee intake was associated with lower CVD risk in males but not females. Conversely, in a prospective study of 7,170 diabetic women, higher caffeine-containing coffee intake was not associated with cardiovascular or all-cause mortality risk [30]. The differences in these studies may be due to different population characteristics, sample sizes, adjustment factors, and follow-up times.

Diabetes and prediabetes are often accompanied by a series of cardiovascular metabolic abnormalities, including insulin resistance, oxidative stress, and lipid metabolism disorder. These are also significant risk factors for CVD. However, it is still unclear whether caffeine affects overall mortality and cardiovascular outcomes in this specific population. Nevertheless, some in vitro and animal experiments suggest that caffeine is a rich source of antioxidants, and there is evidence that coffee is negatively correlated with the risk of cardiovascular metabolic disorders, including CVD, type 2 diabetes, dyslipidemia, and hypertension [31]. Previous studies have found that caffeine can induce thermogenesis and weight loss [32], thus improving insulin sensitivity and glucose-induced insulin secretion, preventing hyperglycemia and oxidative stress.

Moreover, caffeine can alleviate inflammatory reactions and positively affect the prevention and treatment of inflammation-related diseases [33]. It reduces the activity of the critical inflammatory factor NFκB and negatively affects the production of pro-inflammatory cytokines by influencing adenosine receptors. It is worth noting that high doses of caffeine (different from moderate doses) may decrease the production of the anti-inflammatory factor IL-10. This is related to the non-selective antagonism of adenosine A1R and A2R receptors [34]. This observation supports the overall recommendation of moderate caffeine intake in this study. Our research results show that the risk of overall mortality is most significantly reduced when caffeine intake is at 254 mg/d, and as the intake increases, this benefit decreases and even becomes a risk factor.

As the primary source of caffeine, we also need to consider the other effects of coffee and tea because people consume caffeine in the form of beverages. They contain hundreds of phytochemically derived compounds that may have additive and synergistic effects, including chlorogenic acids/lignans, alkaloids, polyphenols, terpenes, melanoids, vitamins, and metals [35]. Several phenols in coffee have the effect of scavenging free radicals [36]. Previous evidence has shown that many components of coffee, including chlorogenic acid, phenols, caffeine, cafestol, and kahweol, are inducers of the nuclear factor erythroid 2–related factor 2(Nrf2), thus contributing to coffee’s important role in alleviating oxidative stress [37–39]. However, a large body of evidence from animal and human studies shows that because the concentration of coffee components is too low, the ability to remove free radicals in the body is limited and may not have much effect on coffee’s health. Polyphenols not only have strong antioxidant properties but may also reduce the activation of pro-inflammatory factors, such as nuclear factor kappa-β (NF-Kβ), and the degree of this reduction in activation is proportional to the concentration of chlorogenic acid (CGA). Similarly, Zhao et al. [40]showed that IL-8 secretion induced by H2O2 or tumor necrosis factor receptor activation can be blocked by 5-O-caffeoyl quinic acid (5-CQA) in CGA in a dose-dependent manner in human intestinal epithelial CaCo2 cells.

Diterpenes are fatty acyl ester compounds that have also attracted attention as bioactive compounds in coffee [41]. The most studied is kahweol, which has been shown to be a potent inhibitor of cell viability in vitro. Similarly,> both kahweol and caffeol (another diterpene) significantly inhibited pro-inflammatory cyclooxygenase-2 (COX-2) protein and its mRNA expression in a dose-dependent manner [42]. There are few studies on the effects of diterpenoids on humans, but there have been reports of an increase in serum total cholesterol [43]. However, further studies still need to confirm the effects of differences in the dose required for enzyme induction on humans. Another interesting phenomenon is that the Maillard reaction is the primary chemical reaction that occurs during the roasting of coffee. Melanoids are produced during coffee roasting as a result of the Maillard reaction. Many studies have confirmed melanoids to have a variety of biological activities, such as hypoglycemic, anti-inflammatory, antioxidant, and anti-tumor [44]. However, this biological property also depends on roasting conditions. It is worth mentioning that melanoids are not digested in the human body and appear as dietary fiber, which may be an essential contributor to colon health. In recent years, more and more studies have focused on the impact of gut microbes on gut health [45, 46]. Studies have shown that coffee regulates gut flora and microbial metabolites. For example, the indigestible polysaccharides in coffee are rapidly metabolized into short-chain fatty acids in the gut, which leads to increased levels of Bacteroides/Prevotella [47]. Another study also reported higher levels of Bacteroide-Prevotella-porphyromonas in people who drank much coffee [48], as well as lower levels of lipid peroxidation and increased microbial production of short-chain fatty acids, which have a chemical protective effect in the gut. The results of several other studies have shown that coffee can induce bacterial species and their metabolites, which are beneficial to the human body [49]. However, the mechanism of action of coffee on changes in the gut microbiome is still unclear.

Our research results indicate that the association between caffeine intake and all-cause mortality and CVD mortality varies by diabetes status (diabetes or prediabetes). In prediabetic patients, caffeine intake is associated with a more significant reduction in the risk of all-cause mortality (26% vs. 18%). The lower risk of CVD mortality associated with caffeine intake is only observed in the prediabetic population, and this association is not found in diabetic individuals. Many prospective cohort studies have reported a negative correlation between increased coffee consumption and the risk of developing T2DM [50]. According to a recent meta-analysis, for every 200 mg/day increase in caffeine intake, the incidence of T2DM decreases by 14% [51]. The protective effect of coffee on T2DM and metabolic syndrome has also been observed in cross-sectional studies [52, 53]. Currently, there is no complete explanation for the biological mechanisms of the differences in caffeine intake and the risk of all-cause mortality and CVD mortality among diabetes populations at different stages. Additionally, studies have indicated that caffeinated coffee and caffeine adversely affect glucose metabolism, which may even occur after several days of continuous coffee consumption. Van Dam et al. [54] found that consuming a high amount of coffee for four consecutive weeks increased fasting insulin levels compared to not drinking coffee. Explaining the similar protective effects of caffeinated beverages on T2DM incidence is challenging if decaffeinated coffee and caffeinated coffee have different metabolic effects. However, this could partially explain the weaker relationship between caffeine intake and long-term all-cause mortality in diabetic populations. Another explanation is that the observed association between coffee and T2DM in observational studies may be spurious rather than causal, as dietary restrictions occur after developing diabetes, including avoiding sugary beverages like coffee and caffeinated drinks. Our analysis of baseline data for prediabetes and diabetes groups also suggests that caffeine intake is significantly higher in the prediabetes population compared to the diabetes population, among other dietary intakes (sugar, fat, protein, etc.).

Currently, there are many prediabetic patients, and diabetic patients, as a high-risk group for diabetes, generally receive less attention. Prediabetes is an asymptomatic chronic mild hyperglycemic state, which, if left undetected, may progress to diabetes [55]. Dietary intake is an essential and modifiable intervention factor for diabetes, and early intervention can improve prognosis. However, prediabetic patients may also be restricted in their diet, such as reducing sugar and carbohydrate intake. The results of this study show a U-shaped association between caffeine intake and the risk of all-cause mortality, emphasizing the guiding role of moderate caffeine intake.

This study has some limitations. Firstly, although the survey conducted long-term follow-up for all included patients, the possibility of residual confounding cannot be ruled out due to the nature of observational studies. Secondly, caffeine intake levels were self-reported at baseline, which may reflect a different level over long-term follow-up. However, this study used the average of two recorded measurements to reflect long-term dietary patterns as much as possible. Thirdly, we did not clearly explain the sources of caffeine, such as coffee, beverages, etc. Fourthly, similar to most observational studies, despite conducting multiple sensitivity analyses, unmeasured confounders (such as mental illness and sleep patterns) may still introduce bias. Fifthly, NHANES data differentiate the types of diabetes, and we included participants aged 20 and above, so the results of this study may be more representative of the population with type 2 diabetes.

## Conclusions

In summary, our research has found that within a certain range, increased caffeine intake is associated with reduced all-cause mortality in patients with diabetes and those with pre-diabetes. In patients with prediabetes, there is also a significant negative correlation between caffeine intake and CVD events, but this association is not present in patients with diabetes.

### Electronic supplementary material

Below is the link to the electronic supplementary material.

Supplementary Material 1

## Data Availability

No datasets were generated or analysed during the current study.
