# Supplementary material for: Association of caffeine intake with all-cause and cardiovascular mortality in diabetes and prediabetes
Source: Diabetol Metab Syndr. 2024 Jul 26;16:177. doi: 10.1186/s13098-024-01417-6 (PMC11282651; doi:10.1186/s13098-024-01417-6)
Supplement: Supplementary file 1 — Supplementary Material 1 [file 13098_2024_1417_MOESM1_ESM.docx]

**Additional Materials**

**Associations of Caffeine Intake With Risk of Cardiovascular Mortality Among Individuals With Prediabetes and Diabetes**

**Haipeng Yao, Lamei Li, Xiabo Wang, Zhongqun Wang**

**Figure S1.** **Directed Acyclic Graph**

**Figure S2. Proportion plot of covariate missing values**

**Table S1. Baseline characteristics of participants with quartiles of caffeine intake among diabetes and prediabetes participants (participants with missing values excluded)**

**Table S2. Baseline characteristics of patients with diabetes and prediabetes in NHANES 2003-2018**

**Table S3. Multivariable-adjusted analysis of caffeine intake associated with all-cause mortality and cardiovascular mortality in participants with no baseline covariator missing values in NHANES 2003-2018**

**Table S4. Multivariable-adjusted analysis of caffeine intake associated with all-cause mortality and cardiovascular mortality among participants who did not died within 2 years of follow-up in NHANES 2003-2018**

**Table S5. Multivariable-adjusted analysis of caffeine intake in relation to all-cause mortality and cardiovascular mortality among participants without baseline cardiovascular disease or cancer in NHANES 2003-2018**

**Table S6. Multivariable-adjusted analysis of caffeine intake associated with all-cause mortality and cardiovascular mortality among insulin therapy and oral hypoglycemic drugs participants in NHANES 2003-2018**

**Table S7. Multivariable-adjusted analysis of caffeine intake associated with all-cause mortality and cardiovascular mortality among diabetic patients in NHANES 2003-2018**

**Figure S3. Multivariable adjusted restricted cubic splines forassociations of caffeine intake with all-cause and cardiovascular mortality among diabetic patients from NHANES 2003-2018**

**Table S8. Multivariable-adjusted analysis of caffeine intake associated with all-cause mortality and cardiovascular mortality among prediabetic patients in NHANES 2003-2018**

**Figure S4. Multivariable adjusted restricted cubic splines forassociations of caffeine intake with all-cause and cardiovascular mortality among prediabetic patients from NHANES 2003-2018**

**Table S9. Multivariate adjustment analysis of caffeine intake associated with all-cause mortality and cardiovascular mortality in participants including LDL-C, TG, and fasting glucose in NHANES 2003-2018.**

**Table S10.** **Baseline characteristics of the lowest and extreme quartiles of caffeine intake after propensity score matching**

**Figure S5.** **Covariate balance between lowest and extreme quartile individuals before and after propensity score matching**

**Table S11. Multivariable-adjusted analysis of caffeine intake associated with all-cause mortality and cardiovascular mortality after propensity score matching**

**Table S12.** **Multivariable-adjusted analysis of coffee consumption associated with all-cause mortality and cardiovascular mortality in patients with diabetes and prediabetes in NHANES 2003-2018**

**
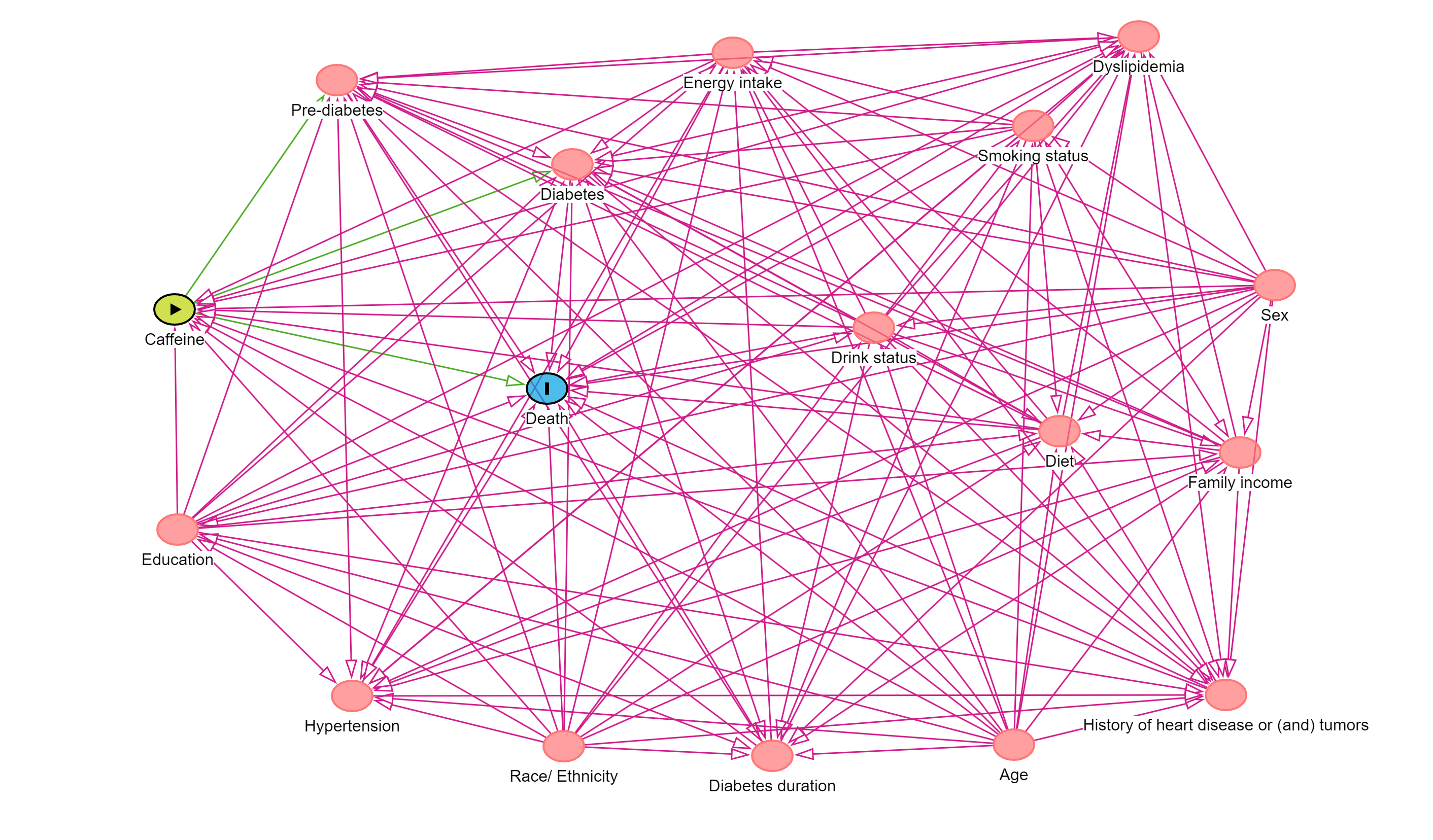
**

**Figure S1.** D**irected Acyclic Graph.** A directed acyclic graph represents the associations between covariates, main exposures, and outcomes.

**
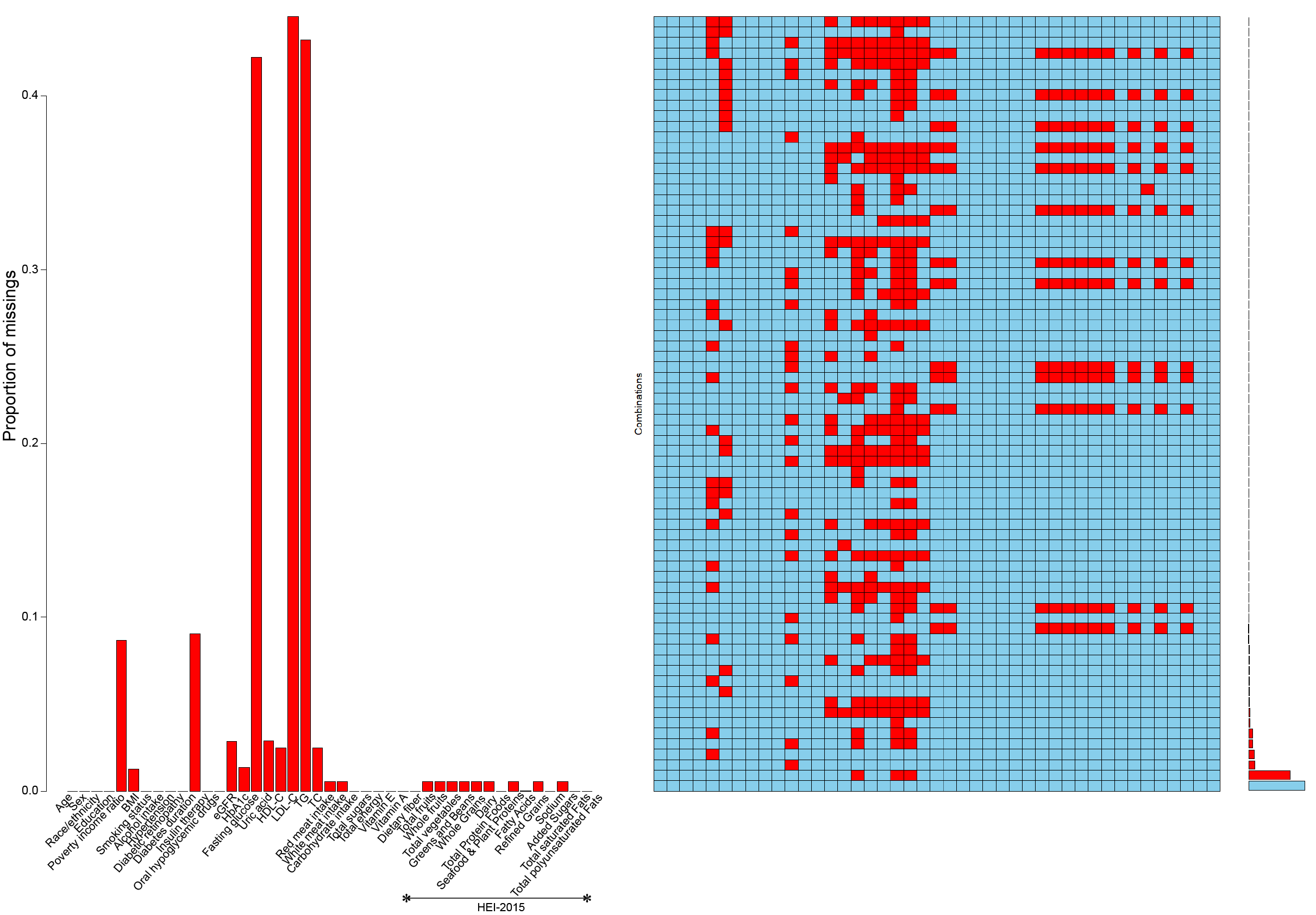
**

**Figure S2. Proportion plot of covariate missing values.** Based on the 13 dietary components mentioned in the 2015-2020 Dietary Guidelines for Americans (DGA), the HEI-2015 scoring system indicates overall dietary quality. BMI: body mass index. HDL-C: HDL cholesterol. LDL-C: LDL cholesterol. TC: total cholesterol.

**Table S1. Baseline characteristics of participants with quartiles of caffeine intake among diabetes and prediabetes participants (participants with missing values excluded).**

| **Characteristic** | **Caffeine intake(mg/d)** | | | | | ***P*** |
| --- | --- | --- | --- | --- | --- | --- |
|  | **Total** | **Quartile 1** ≤32.0 | **Quartile 2** 32.1-103.0 | **Quartile 3**  103.1-209.0 | **Quartile 4** >209.0 |  |
| Patients, n | 14473 | 3621 | 3631 | 3603 | 3618 |  |
| Age, years | 54.0 ± 15.6 | 53.0 ± 17.5 | 52.5 ± 16.5 | 54.3 ± 15.9 | 55.6 ± 13.2 | <0.001 |
| Sex,n(%) |  |  |  |  |  | <0.001 |
| Male | 7,581 (51.7%) | 1,720 (47.1%) | 1,762 (45.7%) | 1,886 (49.3%) | 2,213 (60.6%) |  |
| Female | 6,892(48.3%) | 1,901 (52.9%) | 1,869 (54.3%) | 1,717 (50.7%) | 1,405 (39.4%) |  |
| Race/ethnicity,n(%) |  |  |  |  |  | <0.001 |
| Non-Hispanic White | 6,103 (66.5%) | 1,043 (50.6%) | 1,127 (55.1%) | 1,586 (68.8%) | 2,347 (82.7%) |  |
| Non-Hispanic Black | 3,222 (11.9%) | 1,256 (23.3%) | 963 (16.2%) | 675 (9.4%) | 328 (3.8%) |  |
| Mexican American | 2,406 (8.4%) | 639 (11.2%) | 723 (11.8%) | 628 (8.4%) | 416 (4.2%) |  |
| Other | 2,742 (13.2%) | 683 (14.9%) | 818 (16.9%) | 714 (13.5%) | 527 (9.3%) |  |
| Education level ,n(%) |  |  |  |  |  | <0.001 |
| Less than high | 3,954 (17.9%) | 1,150 (23.2%) | 1,092 (20.3%) | 941 (16.4%) | 771 (13.9%) |  |
| High school grad or equivalent | 3,437 (25.2%) | 819 (23.2%) | 884 (27.3%) | 838 (25.7%) | 896 (24.7%) |  |
| College or above | 7,082 (56.9%) | 1,652 (53.6%) | 1,655 (52.4%) | 1,824 (57.9%) | 1,951 (61.4%) |  |
| Family income-poverty ratio,n(%) |  |  |  |  |  | <0.001 |
| ≤1.30 | 4,468 (21.4%) | 1,307 (28.3%) | 1,210 (25.4%) | 1,036 (20.2%) | 915 (15.1%) |  |
| 1.3-3.5 | 5,687 (37.0%) | 1,411 (38.4%) | 1,466 (39.9%) | 1,432 (35.9%) | 1,378 (34.8%) |  |
| >3.5 | 4,318 (41.7%) | 903 (33.3%) | 955 (34.7%) | 1,135 (43.9%) | 1,325 (50.0%) |  |
| BMI, Kg/m2 |  |  |  |  |  | <0.001 |
| < 25.0 | 2,879 (19.6%) | 771 (21.9%) | 741 (20.6%) | 702 (19.5%) | 665 (17.6%) |  |
| 25.0-29.9 | 4,838 (32.7%) | 1,141 (29.5%) | 1,220 (30.3%) | 1,222 (32.7%) | 1,255 (36.3%) |  |
| ≥30 | 6,756 (47.7%) | 1,709 (48.6%) | 1,670 (49.1%) | 1,679 (47.8%) | 1,698 (46.0%) |  |
| Smoking status ,n(%) |  |  |  |  |  | <0.001 |
| Never | 7,455 (50.8%) | 2,254 (63.4%) | 2,151 (60.2%) | 1,792 (50.4%) | 1,258 (36.6%) |  |
| Former smoker | 4,256 (30.0%) | 905 (24.3%) | 954 (25.4%) | 1,139 (32.7%) | 1,258 (34.6%) |  |
| Current smoker | 2,762 (19.2%) | 462 (12.3%) | 526 (14.4%) | 672 (16.9%) | 1,102 (28.7%) |  |
| Alcohol intake ,n(%) |  |  |  |  |  | <0.001 |
| Never | 10,786 (71.1%) | 2,812 (75.4%) | 2,782 (74.5%) | 2,631 (69.0%) | 2,561 (67.6%) |  |
| Moderate | 2,129 (16.3%) | 436 (12.9%) | 521 (15.1%) | 584 (18.0%) | 588 (17.9%) |  |
| Heavy | 1,558 (12.6%) | 373 (11.6%) | 328 (10.4%) | 388 (13.0%) | 469 (14.5%) |  |
| Hypertension ,n(%) |  |  |  |  |  | 0.12 |
| No | 6,549 (48.2%) | 1,558 (45.9%) | 1,681 (48.7%) | 1,662 (50.1%) | 1,648 (47.9%) |  |
| Yes | 7,924 (51.8%) | 2,063 (54.1%) | 1,950 (51.3%) | 1,941 (49.9%) | 1,970 (52.1%) |  |
| Insulin therapy ,n(%) |  |  |  |  |  | >0.9 |
| No | 13,468 (94.1%) | 3,355 (94.1%) | 3,394 (93.8%) | 3,349 (94.3%) | 3,370 (94.1%) |  |
| Yes | 1,005 (5.9%) | 266 (5.9%) | 237 (6.2%) | 254 (5.7%) | 248 (5.9%) |  |
| Oral hypoglycemic drugs,n(%) |  |  |  |  |  | 0.2 |
| No | 12,363 (87.5%) | 3,118 (88.8%) | 3,105 (87.6%) | 3,039 (87.6%) | 3,101 (86.4%) |  |
| Yes | 2,110 (12.5%) | 503 (11.2%) | 526 (12.4%) | 564 (12.4%) | 517 (13.6%) |  |
| Diabetic retinopathy ,n(%) |  |  |  |  |  | 0.072 |
| No | 13,717 (96.0%) | 3,419 (95.9%) | 3,429 (95.4%) | 3,433 (96.8%) | 3,436 (95.9%) |  |
| Yes | 756 (4.0%) | 202 (4.1%) | 202 (4.6%) | 170 (3.2%) | 182 (4.1%) |  |
| Diabetes | 3,774 (22.2%) | 959 (22.1%) | 937 (22.4%) | 957 (21.6%) | 921 (22.6%) | 0.9 |
| Prediabetes | 10,699 (77.8%) | 2,662 (77.9%) | 2,694 (77.6%) | 2,646 (78.4%) | 2,697 (77.4%) | 0.9 |
| Diabetes duration |  |  |  |  |  | >0.9 |
| ≤3 | 11,620 (83.7%) | 2,919 (84.6%) | 2,934 (83.6%) | 2,857 (83.5%) | 2,910 (83.3%) |  |
| 3-10 | 1,250 (7.4%) | 303 (7.1%) | 319 (7.5%) | 317 (7.3%) | 311 (7.8%) |  |
| >10 | 1,603 (8.9%) | 399 (8.3%) | 378 (8.8%) | 429 (9.2%) | 397 (8.9%) |  |
| HbA_1c_(%) |  |  |  |  |  | 0.08 |
| <7 | 12,582 (89.4%) | 3,162 (90.5%) | 3,143 (87.9%) | 3,114 (89.7%) | 3,163 (89.7%) |  |
| ≥7 | 1,891 (10.6%) | 459 (9.5%) | 488 (12.1%) | 489 (10.3%) | 455 (10.3%) |  |
| eGFR(mL/min/1.73m2) | 90.3 ± 21.0 | 90.3 ± 22.9 | 91.2 ± 22.7 | 90.8 ± 20.9 | 89.3 ± 18.5 | 0.002 |
| Uric acid(mg/dL) | 5.7 ± 1.4 | 5.6 ± 1.5 | 5.7 ± 1.4 | 5.6 ± 1.4 | 5.8 ± 1.4 | 0.009 |
| HDL-C (mg/dL) | 51.2 ± 15.3 | 51.8 ± 15.6 | 50.6 ± 14.9 | 52.0 ± 15.7 | 50.6 ± 15.0 | 0.001 |
| TC(mg/dL) | 197.3 ± 43.1 | 192.9 ± 42.8 | 198.4 ± 44.3 | 198.0 ± 42.6 | 198.8 ± 42.8 | <0.001 |
| HEI-2015 | 49.9 ± 11.9 | 52.0 ± 12.4 | 49.8 ± 11.9 | 49.8 ± 11.9 | 48.8 ± 11.6 | <0.001 |
| Total energy(Kcal/d) | 1,942.0 (1,512.5, 2,477.0) | 1,796.0 (1,386.3, 2,288.0) | 1,834.8 (1,405.0, 2,326.0) | 1,951.8 (1,527.3, 2,480.5) | 2,101.0 (1,669.0, 2,669.5) | <0.001 |
| Total carbohydrate intake(g/d) | 226.6 (173.5, 295.8) | 215.9 (162.2, 275.9) | 221.7 (166.9, 281.1) | 226.2 (174.8, 299.2) | 237.5 (185.6, 310.1) | <0.001 |
| Total protein(g/d) | 76.2 (58.0, 98.1) | 72.8 (55.5, 93.7) | 72.5 (55.4, 92.9) | 75.4 (57.2, 97.9) | 82.1 (62.4, 104.6) | <0.001 |
| Total sugars (g/d) | 92.8 (62.9, 134.3) | 84.8 (57.6, 122.5) | 91.9 (63.5, 128.4) | 92.3 (63.4, 138.4) | 98.1 (64.6, 142.8) | <0.001 |
| Dietary fiber(g/d) | 15.4 (10.7, 21.2) | 15.2 (10.8, 21.5) | 14.5 (10.0, 20.6) | 15.3 (10.9, 20.6) | 16.0 (11.2, 21.6) | 0.001 |
| Total saturated fatty acids (g/d) | 24.1 (16.8, 33.4) | 21.0 (14.1, 30.4) | 21.8 (15.3, 30.1) | 24.4 (17.4, 33.1) | 27.5 (19.7, 37.2) | <0.001 |
| Total polyunsaturated fatty acids (g/d) | 16.4 (11.3, 23.1) | 15.1 (10.0, 21.0) | 15.2 (10.4, 21.8) | 16.7 (11.5, 23.4) | 18.1 (12.7, 24.9) | <0.001 |
| Vitamin E(mg/d) | 7.1 (4.9, 10.1) | 6.6 (4.5, 9.3) | 6.6 (4.5, 9.5) | 7.2 (5.0, 10.2) | 7.8 (5.3, 10.8) | <0.001 |
| Vitamin A(mg/d) | 0.5 (0.3-0.8) | 0.5（0.3-0.8） | 0.5（0.3-0.8） | 0.5（0.3-0.8） | 0.6（0.4-0.8） | <0.001 |
| Red meat intake(oz. eq./d) | 1.1 (0.0, 2.6) | 0.9 (0.0, 2.3) | 1.1 (0.0, 2.4) | 1.2 (0.0, 2.6) | 1.3 (0.0, 2.8) | <0.001 |
| White meat intake(oz. eq./d) | 0.8 (0.0, 2.3) | 1.0 (0.0, 2.4) | 0.8 (0.0, 2.3) | 0.8 (0.0, 2.2) | 0.8 (0.0, 2.2) | 0.088 |

Continuous variables with a normal distribution are described as the mean ± standard deviation (SD), while continuous variables with a non-normal distribution are described as the median (interquartile range). Categorical variables are represented by numbers (weighted percentages). BMI: body mass index. HDL-C: HDL cholesterol. TC: total cholesterol.

**Table S2. Baseline characteristics of patients with diabetes and prediabetes in NHANES 2003-2018.**

| **Characteristic** | **Total** | **Prediabetes** | **Diabetes** | ***P*** |
| --- | --- | --- | --- | --- |
| Patients, n | 18914 | 12390 | 6524 |  |
| Age, years | 54.8 ± 15.7 | 52.8 ± 16.1 | 59.7 ± 13.6 | <0.001 |
| Sex,n(%) |  |  |  | 0.800 |
| Male | 9,746 (51.0%) | 6,396 (50.9%) | 3,350 (51.2%) |  |
| Female | 9,168 (49.0%) | 5,994 (49.1%) | 3,174 (48.8%) |  |
| Race/ethnicity,n(%) |  |  |  | <0.001 |
| Non-Hispanic White | 7,652 (64.8%) | 5,295 (66.4%) | 2,357 (61.0%) |  |
| Non-Hispanic Black | 4,401 (12.9%) | 2,693 (11.9%) | 1,708 (15.3%) |  |
| Mexican American | 3,203 (8.8%) | 1,965 (8.5%) | 1,238 (9.7%) |  |
| Other | 3,658 (13.5%) | 2,437 (13.3%) | 1,221 (14.1%) |  |
| Education level ,n(%) |  |  |  | <0.001 |
| Less than high | 5,489 (19.1%) | 3,249 (17.1%) | 2,240 (24.1%) |  |
| High school grad or equivalent | 4,488 (25.4%) | 2,947 (25.3%) | 1,541 (25.8%) |  |
| College or above | 8,937 (55.4%) | 6,194 (57.7%) | 2,743 (50.1%) |  |
| Family income-poverty ratio ,n(%) |  |  |  | <0.001 |
| ≤1.30 | 6,045 (22.8%) | 3,753 (21.3%) | 2,292 (26.4%) |  |
| 1.3-3.5 | 7,426 (37.3%) | 4,763 (36.3%) | 2,663 (39.5%) |  |
| >3.5 | 5,443 (39.9%) | 3,874 (42.4%) | 1,569 (34.0%) |  |
| BMI, Kg/m2 |  |  |  | <0.001 |
| < 25.0 | 3,638 (18.8%) | 2,777 (21.8%) | 861 (11.8%) |  |
| 25.0-29.9 | 6,138 (31.6%) | 4,320 (34.4%) | 1,818 (25.1%) |  |
| ≥30 | 9,138 (49.5%) | 5,293 (43.9%) | 3,845 (63.0%) |  |
| Smoking status ,n(%) |  |  |  | <0.001 |
| Never | 9,749 (50.7%) | 6,505 (51.4%) | 3,244 (48.9%) |  |
| Former smoker | 5,592 (30.1%) | 3,372 (28.3%) | 2,220 (34.4%) |  |
| Current smoker | 3,573 (19.2%) | 2,513 (20.2%) | 1,060 (16.6%) |  |
| Alcohol intake ,n(%) |  |  |  | <0.001 |
| Never | 14,335 (72.4%) | 8,990 (69.2%) | 5,345 (80.0%) |  |
| Moderate | 2,666 (15.6%) | 1,919 (17.0%) | 747 (12.4%) |  |
| Heavy | 1,913 (12.0%) | 1,481 (13.8%) | 432 (7.6%) |  |
| Hypertension ,n(%) |  |  |  | <0.001 |
| No | 8,223 (46.9%) | 6,393 (54.2%) | 1,830 (29.5%) |  |
| Yes | 10,691 (53.1%) | 5,997 (45.8%) | 4,694 (70.5%) |  |
| Insulin therapy ,n(%) |  |  |  |  |
| No | - | - | 5,160 (79.4%) |  |
| Yes | - | - | 1,364 (20.6%) |  |
| Oral hypoglycemic drugs ,n(%) |  |  |  |  |
| No | - | - | 3,602 (55.2%) |  |
| Yes | - | - | 2,922 (44.8%) |  |
| Diabetic retinopathy ,n(%) |  |  |  |  |
| No | - | - | 5,500 (86.0%) |  |
| Yes | - | - | 1,024 (14.0%) |  |
| Diabetes duration |  |  |  |  |
| ≤3 | - | - | 1,749 (29.1%) |  |
| 3-10 | - | - | 2,106 (32.9%) |  |
| >10 | - | - | 2,669 (38.0%) |  |
| HbA_1c_(%) |  |  |  |  |
| <7 | - | - | 3,611 (58.0%) |  |
| ≥7 | - | - | 2,913 (42.0%) |  |
| eGFR(mL/min/1.73m2) | 89.6 ± 21.7 | 91.8 ± 20.3 | 84.5 ± 23.9 | <0.001 |
| Uric acid(mg/dL) | 5.7 ± 1.4 | 5.7 ± 1.4 | 5.8 ± 1.5 | 0.069 |
| HDL-Cholesterol (mg/dL) | 50.9 ± 15.5 | 52.3 ± 15.6 | 47.6 ± 14.5 | <0.001 |
| TC(mg/dL) | 197.1 ± 43.8 | 201.0 ± 41.2 | 187.8 ± 48.3 | <0.001 |
| HEI-2015 | 49.9 ± 11.9 | 49.8 ± 12.1 | 50.2 ± 11.5 | 0.025 |
| Total energy(Kcal/d) | 1,912.0 (1,481.0, 2,442.7) | 1,970.5 (1,536.5, 2,510.2) | 1,782.5 (1,376.1, 2,271.7) | <0.001 |
| Total carbohydrate intake(g/d) | 224.7 (170.5, 292.1) | 232.5 (178.1, 302.2) | 206.9 (156.3, 265.0) | <0.001 |
| Total protein(g/d) | 75.2 (57.1, 97.3) | 76.4 (58.1, 98.4) | 72.1 (54.6, 93.5) | <0.001 |
| Total sugars (g/d) | 92.0 (62.2, 133.3) | 97.4 (66.5, 140.9) | 79.8 (53.7, 113.7) | <0.001 |
| Dietary fiber(g/d) | 15.1 (10.7, 20.8) | 15.3 (10.7, 21.2) | 14.8 (10.6, 20.1) | 0.013 |
| Total saturated fatty acids (g/d) | 23.6 (16.4, 32.8) | 24.2 (16.9, 33.6) | 22.3 (15.5, 31.2) | <0.001 |
| Total polyunsaturated fatty acids (g/d) | 16.1 (11.0, 22.7) | 16.4 (11.3, 23.0) | 15.3 (10.5, 21.9) | <0.001 |
| Vitamin E(mg/d) | 6.9 (4.8, 9.9) | 7.1 (4.9, 10.1) | 6.5 (4.5, 9.3) | <0.001 |
| Vitamin A(mg/d) | 0.5 (0.3-0.8) | 0.5（0.3-0.8） | 0.5（0.3-0.8） | 0.300 |
| Red meat intake(oz. eq./d) | 1.1 (0.0, 2.5) | 1.1 (0.0, 2.6) | 1.1 (0.0, 2.4) | 0.200 |
| White meat intake(oz. eq./d) | 0.8 (0.0, 2.3) | 0.9 (0.0, 2.3) | 0.7 (0.0, 2.1) | 0.002 |
| Caffeine intake(mg) | 125 (42, 242) | 129 (43, 246) | 118 (39, 234) | 0.012 |

Continuous variables with a normal distribution are described as the mean ± standard deviation (SD), while continuous variables with a non-normal distribution are described as the median (interquartile range). Categorical variables are represented by numbers (weighted percentages). BMI: body mass index. HDL-C: HDL cholesterol. TC: total cholesterol.

**Table S3. Multivariable-adjusted analysis of caffeine intake associated with all-cause mortality and cardiovascular mortality in participants with no baseline covariator missing values in NHANES 2003-2018**

|  | **Quartiles of caffeine intake (mg/d)** | | | | ***P* for trend** |
| --- | --- | --- | --- | --- | --- |
| **Categories** | **Quartile 1 ≤32.0** | **Quartile 2 32.1-103.0** | **Quartile 3 103.1-209.0** | **Quartile 4 >209.0** |  |
| **All-cause mortality** |  |  |  |  |  |
| No. deaths/total | 586/3621 | 528/3631 | 506/3603 | 549/3618 |  |
| Crude | 1 | 0.82(0.71-0.96) | 0.77(0.64-0.92) | 0.86(0.72-1.02) | 0.330 |
| Model1 | 1 | 0.95(0.81-1.12) | 0.81(0.68-0.96) | 0.88(0.74-1.06) | 0.193 |
| Model2 | 1 | 0.94(0.80-1.1) | 0.8(0.67-0.94) | 0.87(0.72-1.05) | 0.156 |
| Model3 | 1 | 0.92(0.78-1.08) | 0.78(0.66-0.93) | 0.86(0.71-1.03) | 0.144 |
| **CVD mortality** |  |  |  |  |  |
| No. deaths/total | 201/3621 | 193/3631 | 161/3603 | 167/3618 |  |
| Crude | 1 | 0.91(0.72-1.16) | 0.77(0.58-1.02) | 0.79(0.61-1.04) | 0.150 |
| Model1 | 1 | 1.06(0.83-1.36) | 0.86(0.65-1.14) | 0.93(0.70-1.23) | 0.470 |
| Model2 | 1 | 1.04(0.81-1.35) | 0.84(0.64-1.11) | 0.9(0.68-1.19) | 0.367 |
| Model3 | 1 | 1.02(0.79-1.32) | 0.83(0.63-1.09) | 0.89(0.66-1.19) | 0.366 |

Model 1: adjusted for sex (female or male), age (continuous), race/ethnicity (non-Hispanic white, non-Hispanic black, Mexican American, and other races), BMI (<25.0, 25.0-29.9, ≥30), education level (less than high school, high school or equivalent, university or higher), family income-to-poverty ratio (≤1.3, 1.3-3.5, >3.5), smoking status (never smoked, former smoker, current smoker), alcohol consumption (non-drinker, moderate drinker, heavy drinker), and hypertension (yes or no).

Model 2: Model 1 + eGFR (continuous), HbA1c (<7%, ≥7%), HDL-C (continuous), TC (continuous), and SUA (continuous).

Model 3: Model 2 +HEI-2015 (continuou), red meat intake (oz. eq./d, continuous), white meat intake (oz. eq./d, continuous), total protein (continuous), carbohydrate intake (continuous), total sugars (continuous), total energy (continuous), total saturated fatty acids (continuous), total polyunsaturated fatty acids (continuous), dietary fiber (continuous), vitamin E (continuous), vitamin A (continuous).

**Table S4. Multivariate adjustment analysis of caffeine intake and all-cause and cardiovascular mortality in patients with diabetes and prediabetes after excluding participants died within two years in NHANES 2003-2018.**

| **Categories** | **Quartiles of caffeine intake (mg/d)** | | | | ***P* for trend** |
| --- | --- | --- | --- | --- | --- |
|  | **Quartile 1 ≤29.0** | **Quartile 2 29.1-98.5** | **Quartile 3 98.6-201.0** | **Quartile 4 ＞201.0** |  |
| **All-cause mortality** |  |  |  |  |  |
| No. deaths/total | 554/4447 | 461/4459 | 493/4453 | 524/4421 |  |
| Crude | 1 | 0.73(0.62-0.86) | 0.75(0.63-0.91) | 0.79(0.66-0.95) | 0.154 |
| Model1 | 1 | 0.85(0.71-1.03) | 0.8(0.67-0.95) | 0.84(0.69-1.02) | 0.158 |
| Model2 | 1 | 0.86(0.72-1.03) | 0.79(0.66-0.93) | 0.84(0.69-1.02) | 0.153 |
| Model3 | 1 | 0.85(0.71-1.02) | 0.77(0.65-0.92) | 0.83(0.68-1.01) | 0.152 |
| **CVD mortality** |  |  |  |  |  |
| No. deaths/total | 189/4447 | 160/4459 | 156/4453 | 162/4421 |  |
| Crude | 1 | 0.77(0.57-1.03) | 0.73(0.55-0.98) | 0.7(0.53-0.92) | 0.031 |
| Model1 | 1 | 0.91(0.68-1.22) | 0.83(0.63-1.1) | 0.89(0.66-1.2) | 0.528 |
| Model2 | 1 | 0.92(0.69-1.23) | 0.82(0.62-1.07) | 0.89(0.66-1.2) | 0.507 |
| Model3 | 1 | 0.85(0.71-1.02) | 0.77(0.65-0.92) | 0.83(0.69-1.01) | 0.446 |

Model 1: adjusted for sex (female or male), age (continuous), race/ethnicity (non-Hispanic white, non-Hispanic black, Mexican American, and other races), BMI (<25.0, 25.0-29.9, ≥30), education level (less than high school, high school or equivalent, university or higher), family income-to-poverty ratio (≤1.3, 1.3-3.5, >3.5), smoking status (never smoked, former smoker, current smoker), alcohol consumption (non-drinker, moderate drinker, heavy drinker), and hypertension (yes or no).

Model 2: Model 1 + eGFR (continuous), HbA1c (<7%, ≥7%), HDL-C (continuous), TC (continuous), and SUA (continuous).

Model 3: Model 2 +HEI-2015 (continuou), red meat intake (oz. eq./d, continuous), white meat intake (oz. eq./d, continuous), total protein (continuous), carbohydrate intake (continuous), total sugars (continuous), total energy (continuous), total saturated fatty acids (continuous), total polyunsaturated fatty acids (continuous), dietary fiber (continuous), vitamin E (continuous), vitamin A (continuous).

**Table S5. Multivariable-adjusted analysis of caffeine intake in relation to all-cause mortality and cardiovascular mortality among participants without baseline cardiovascular disease or cancer in NHANES 2003-2018**

| **Categories** | **Quartiles of caffeine intake (mg/d)** | | | | ***P* for trend** |
| --- | --- | --- | --- | --- | --- |
|  | **Quartile 1 ≤29.0** | **Quartile 2 29.1-95.5** | **Quartile 3 95.6-195.0** | **Quartile 4 ＞195.0** |  |
| **All-cause mortality** |  |  |  |  |  |
| No. deaths/total | 399/3552 | 361/3494 | 362/3515 | 385/3508 |  |
| Crude | 1 | 0.79(0.66-0.94) | 0.79(0.65-0.97) | 0.80(0.65-1) | 0.182 |
| Model1 | 1 | 0.85(0.7-1.04) | 0.79(0.64-0.98) | 0.71(0.56-0.9) | 0.008 |
| Model2 | 1 | 0.83(0.68-1.02) | 0.77(0.62-0.95) | 0.7(0.56-0.89) | 0.006 |
| Model3 | 1 | 0.82(0.67-1.01) | 0.76(0.61-0.94) | 0.7(0.55-0.9) | 0.011 |
| **CVD mortality** |  |  |  |  |  |
| No. deaths/total | 127/3552 | 119/3494 | 104/3515 | 101/3508 |  |
| Crude | 1 | 0.77(0.56-1.06) | 0.72(0.52-1.01) | 0.65(0.46-0.93) | 0.044 |
| Model1 | 1 | 0.86(0.62-1.2) | 0.77(0.55-1.09) | 0.66(0.46-0.95) | 0.032 |
| Model2 | 1 | 0.84(0.6-1.17) | 0.76(0.54-1.06) | 0.65(0.45-0.93) | 0.026 |
| Model3 | 1 | 0.82(0.67-1.01) | 0.76(0.61-0.94) | 0.7(0.55-0.9) | 0.038 |

Model 1: adjusted for sex (female or male), age (continuous), race/ethnicity (non-Hispanic white, non-Hispanic black, Mexican American, and other races), BMI (<25.0, 25.0-29.9, ≥30), education level (less than high school, high school or equivalent, university or higher), family income-to-poverty ratio (≤1.3, 1.3-3.5, >3.5), smoking status (never smoked, former smoker, current smoker), alcohol consumption (non-drinker, moderate drinker, heavy drinker), and hypertension (yes or no).

Model 2: Model 1 + eGFR (continuous), HbA1c (<7%, ≥7%), HDL-C (continuous), TC (continuous), and SUA (continuous).

Model 3: Model 2 +HEI-2015 (continuou), red meat intake (oz. eq./d, continuous), white meat intake (oz. eq./d, continuous), total protein (continuous), carbohydrate intake (continuous), total sugars (continuous), total energy (continuous), total saturated fatty acids (continuous), total polyunsaturated fatty acids (continuous), dietary fiber (continuous), vitamin E (continuous), vitamin A (continuous).

**Table S6. Multivariable-adjusted analysis of caffeine intake associated with all-cause mortality and cardiovascular mortality among insulin therapy and oral hypoglycemic drugs participants in NHANES 2003-2018**

| **Categories** | **Quartiles of caffeine intake (mg/d)** | | | | ***P* for trend** |
| --- | --- | --- | --- | --- | --- |
|  | **Quartile 1 ≤29.0** | **Quartile 2 29.1-98.0** | **Quartile 3 98.1-201.5** | **Quartile 4 ＞201.5** |  |
| **All-cause mortality** |  |  |  |  |  |
| No. deaths/total | 641/3824 | 553/3799 | 544/3790 | 555/3790 |  |
| Crude | 1 | 0.75(0.65-0.87) | 0.73(0.62-0.87) | 0.73(0.62-0.87) | 0.008 |
| Model1 | 1 | 0.86(0.74-1.01) | 0.76(0.64-0.9) | 0.77(0.65-0.92) | 0.008 |
| Model2 | 1 | 0.87(0.74-1.01) | 0.76(0.64-0.89) | 0.78(0.65-0.92) | 0.010 |
| Model3 | 1 | 0.84(0.72-0.97) | 0.74(0.62-0.87) | 0.75(0.63-0.90) | 0.007 |
| **CVD mortality** |  |  |  |  |  |
| No. deaths/total | 208/3824 | 194/3799 | 171/3790 | 152/3790 |  |
| Crude | 1 | 0.85(0.68-1.07) | 0.74(0.58-0.95) | 0.66(0.51-0.86) | 0.005 |
| Model1 | 1 | 0.99(0.78-1.27) | 0.82(0.64-1.05) | 0.82(0.63-1.07) | 0.114 |
| Model2 | 1 | 0.99(0.78-1.27) | 0.81(0.63-1.04) | 0.82(0.63-1.07) | 0.121 |
| Model3 | 1 | 0.84(0.72-0.97) | 0.74(0.62-0.87) | 0.75(0.63-0.9) | 0.095 |

Model 1: adjusted for sex (female or male), age (continuous), race/ethnicity (non-Hispanic white, non-Hispanic black, Mexican American, and other races), BMI (<25.0, 25.0-29.9, ≥30), education level (less than high school, high school or equivalent, university or higher), family income-to-poverty ratio (≤1.3, 1.3-3.5, >3.5), smoking status (never smoked, former smoker, current smoker), alcohol consumption (non-drinker, moderate drinker, heavy drinker), and hypertension (yes or no).

Model 2: Model 1 + eGFR (continuous), HbA1c (<7%, ≥7%), HDL-C (continuous), TC (continuous), and SUA (continuous).

Model 3: Model 2 +HEI-2015 (continuou), red meat intake (oz. eq./d, continuous), white meat intake (oz. eq./d, continuous), total protein (continuous), carbohydrate intake (continuous), total sugars (continuous), total energy (continuous), total saturated fatty acids (continuous), total polyunsaturated fatty acids (continuous), dietary fiber (continuous), vitamin E (continuous), vitamin A (continuous), diabetes duration (≤3，3-10，>10), diabetic retinopathy (yes or no).

**Table S7. Multivariable-adjusted analysis of caffeine intake associated with all-cause mortality and cardiovascular mortality among diabetic patients in NHANES 2003-2018**

| **Categories** | **Quartiles of caffeine intake (mg/d)** | | | | ***P* for trend** |
| --- | --- | --- | --- | --- | --- |
|  | **Quartile 1 ≤25.5** | **Quartile 2 25.6-94.2** | **Quartile 3 94.3-189.5** | **Quartile 4 ＞189.5** |  |
| **All-cause mortality** |  |  |  |  |  |
| No. deaths/total | 415/1640 | 351/1622 | 372/1633 | 426/1629 |  |
| Crude | 1 | 0.75(0.63-0.9) | 0.85(0.7-1.03) | 0.80(0.66-0.97) | 0.188 |
| Model1 | 1 | 0.86(0.71-1.04) | 0.91(0.75-1.1) | 0.83(0.68-1.01) | 0.154 |
| Model2 | 1 | 0.84(0.7-1.02) | 0.9(0.75-1.08) | 0.82(0.68-1) | 0.139 |
| Model3 | 1 | 0.85(0.7-1.02) | 0.90(0.75-1.09) | 0.82(0.68-0.99) | 0.121 |
| **CVD mortality** |  |  |  |  |  |
| No. deaths/total | 142/1640 | 115/1622 | 117/1633 | 158/1629 |  |
| Crude | 1 | 0.73(0.52-1.03) | 0.86(0.62-1.22) | 0.92(0.65-1.32) | 0.799 |
| Model1 | 1 | 0.85(0.6-1.2) | 0.97(0.7-1.35) | 1.05(0.75-1.48) | 0.482 |
| Model2 | 1 | 0.84(0.59-1.2) | 0.97(0.71-1.34) | 1.06(0.76-1.47) | 0.408 |
| Model3 | 1 | 0.84(0.59-1.21) | 0.99(0.72-1.38) | 1.08(0.78-1.51) | 0.323 |

Model 1: adjusted for sex (female or male), age (continuous), race/ethnicity (non-Hispanic white, non-Hispanic black, Mexican American, and other races), BMI (<25.0, 25.0-29.9, ≥30), education level (less than high school, high school or equivalent, university or higher), family income-to-poverty ratio (≤1.3, 1.3-3.5, >3.5), smoking status (never smoked, former smoker, current smoker), alcohol consumption (non-drinker, moderate drinker, heavy drinker), and hypertension (yes or no).

Model 2: Model 1 + eGFR (continuous), HbA1c (<7%, ≥7%), HDL-C (continuous), TC (continuous), and SUA (continuous).

Model 3: Model 2 +HEI-2015 (continuou), red meat intake (oz. eq./d, continuous), white meat intake (oz. eq./d, continuous), total protein (continuous), carbohydrate intake (continuous), total sugars (continuous), total energy (continuous), total saturated fatty acids (continuous), total polyunsaturated fatty acids (continuous), dietary fiber (continuous), vitamin E (continuous), vitamin A (continuous), diabetes duration (≤3，3-10，>10), diabetic retinopathy (yes or no), insulin therapy (yes or no), oral hypoglycemic drugs (yes or no).

**
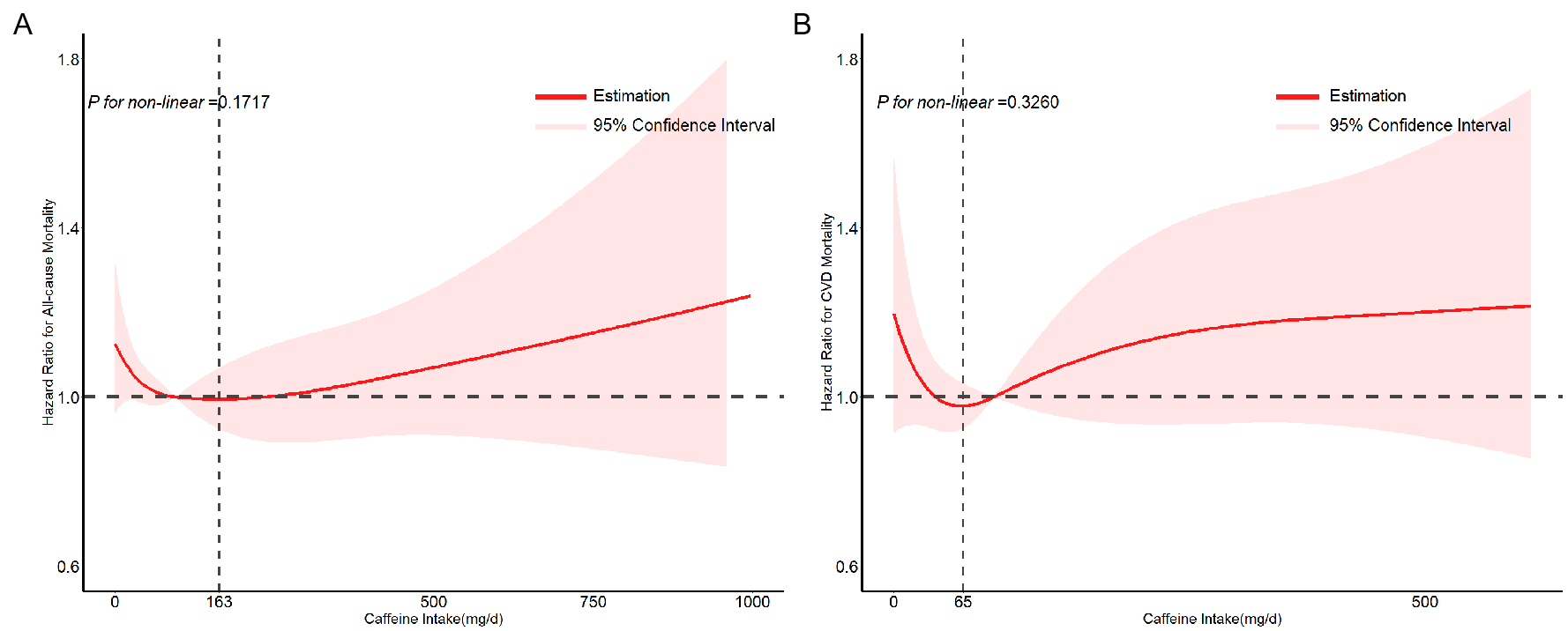
**

**Figure S3. Multivariable adjusted restricted cubic splines forassociations of caffeine intake with all-cause and cardiovascular mortality among diabetic patients from NHANES 2003-2018.** The four nodes of the restricted cubic spline (RCS) are set at the 5th, 35th, 65th, and 95th percentiles of caffeine intake distribution. To determine whether the optimal model is linear or non-linear, we conducted a likelihood ratio test and calculated the p-value for non-linearity. Complete model were adjusted for sex (female or male), age (continuous), race/ethnicity (non-Hispanic white, non-Hispanic black, Mexican American, and other races), BMI (<25.0, 25.0-29.9, ≥30), education level (less than high school, high school or equivalent, university or higher), family income-to-poverty ratio (≤1.3, 1.3-3.5, >3.5), smoking status (never smoked, former smoker, current smoker), alcohol consumption (non-drinker, moderate drinker, heavy drinker), hypertension (yes or no), eGFR (continuous), HbA1c (<7%, ≥7%), HDL-C (continuous), TC (continuous), SUA (continuous), HEI-2015 (continuous), red meat intake (oz. eq./d, continuous), white meat intake (oz. eq./d, continuous), total protein (continuous), carbohydrate intake (continuous), total sugars (continuous), total energy (continuous), total saturated fatty acids (continuous), total polyunsaturated fatty acids (continuous), dietary fiber (continuous), vitamin E (continuous), vitamin A (continuous), diabetes duration (≤3, 3-10, >10), diabetic retinopathy (yes or no), insulin therapy (yes or no), oral hypoglycemic drugs (yes or no). The pale pink shaded area represents a 95% confidence interval.

*A:* Dose-response relationship between caffeine intake and all-cause mortality in diabetic patients in NHANES 2003-2018. *P* for non linear = 0.1717.

*B:* Dose-response relationship between caffeine intake and cardiovascular mortality in diabetic patients in NHANES 2003-2018. *P* for non linear =0.3260.

**Table S8. Multivariable-adjusted analysis of caffeine intake associated with all-cause mortality and cardiovascular mortality among prediabetic patients in NHANES 2003-2018**

| **Categories** | **Quartiles of caffeine intake (mg/d)** | | | | ***P* trend** |
| --- | --- | --- | --- | --- | --- |
|  | **Quartile 1 ≤30.5** | **Quartile 2 30.6-100.5** | **Quartile 3 100.6-205.0** | **Quartile 4 ＞205.0** |  |
| **All-cause mortality** |  |  |  |  |  |
| No. deaths/total | 459/3109 | 383/3100 | 376/3085 | 384/3096 |  |
| Crude | 1 | 0.70(0.59-0.84) | 0.68(0.57-0.82) | 0.73(0.6-0.89) | 0.048 |
| Model1 | 1 | 0.81(0.67-0.98) | 0.7(0.58-0.85) | 0.75(0.62-0.91) | 0.013 |
| Model2 | 1 | 0.82(0.68-0.99) | 0.71(0.58-0.86) | 0.76(0.63-0.92) | 0.016 |
| Model3 | 1 | 0.8(0.67-0.96) | 0.69(0.57-0.83) | 0.74(0.61-0.9) | 0.011 |
| **CVD mortality** |  |  |  |  |  |
| No. deaths/total | 144/3109 | 146/3100 | 115/3085 | 94/3096 |  |
| Crude | 1 | 0.87(0.66-1.15) | 0.65(0.49-0.86) | 0.58(0.41-0.83) | 0.004 |
| Model1 | 1 | 1.02(0.78-1.34) | 0.71(0.53-0.95) | 0.71(0.51-0.99) | 0.026 |
| Model2 | 1 | 1.03(0.78-1.36) | 0.7(0.52-0.94) | 0.71(0.51-0.98) | 0.021 |
| Model3 | 1 | 0.99(0.76-1.3) | 0.68(0.51-0.9) | 0.68(0.48-0.96) | 0.022 |

Model 1: adjusted for sex (female or male), age (continuous), race/ethnicity (non-Hispanic white, non-Hispanic black, Mexican American, and other races), BMI (<25.0, 25.0-29.9, ≥30), education level (less than high school, high school or equivalent, university or higher), family income-to-poverty ratio (≤1.3, 1.3-3.5, >3.5), smoking status (never smoked, former smoker, current smoker), alcohol consumption (non-drinker, moderate drinker, heavy drinker), and hypertension (yes or no).

Model 2: Model 1 + eGFR (continuous), HbA1c (<7%, ≥7%), HDL-C (continuous), TC (continuous), and SUA (continuous).

Model 3: Model 2 +HEI-2015 (continuou), red meat intake (oz. eq./d, continuous), white meat intake (oz. eq./d, continuous), total protein (continuous), carbohydrate intake (continuous), total sugars (continuous), total energy (continuous), total saturated fatty acids (continuous), total polyunsaturated fatty acids (continuous), dietary fiber (continuous), vitamin E (continuous), vitamin A (continuous).

**
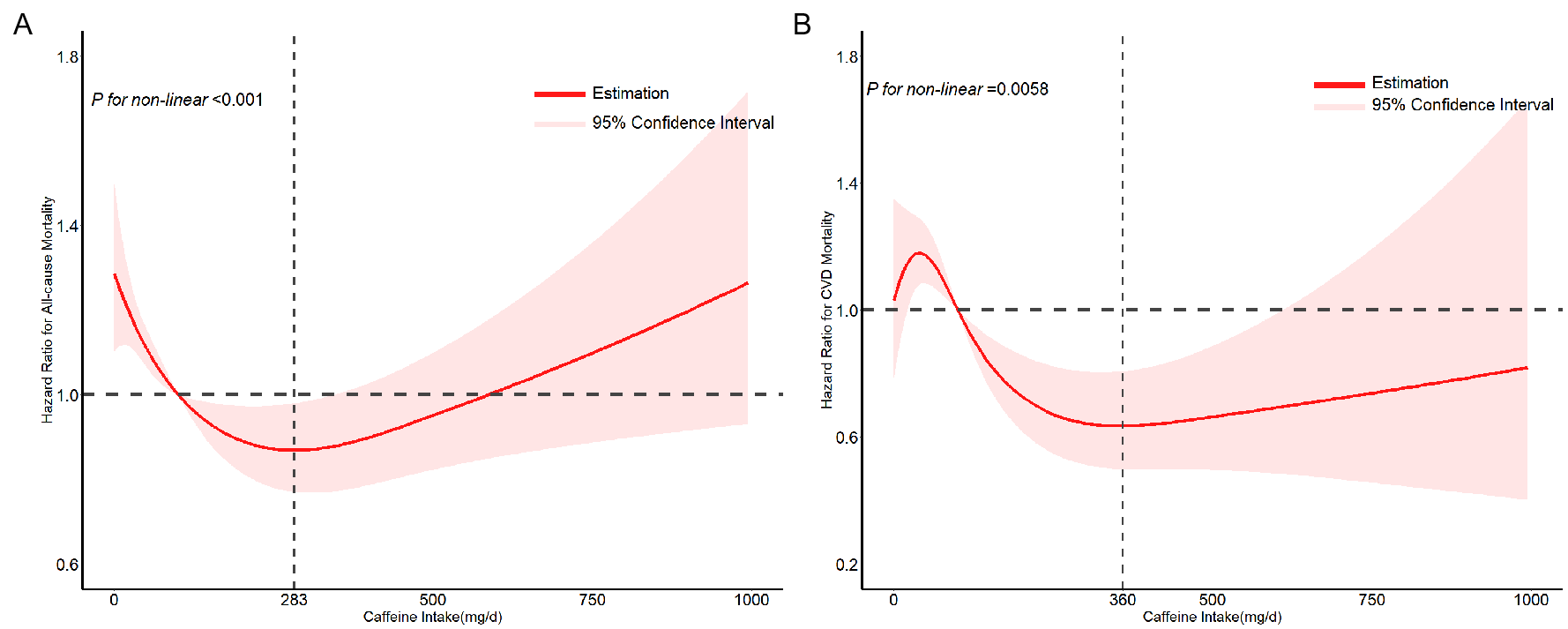
**

**Figure S4. Multivariable adjusted restricted cubic splines forassociations of caffeine intake with all-cause and cardiovascular mortality among prediabetic patients from NHANES 2003-2018.** The four nodes of the restricted cubic spline (RCS) are set at the 5th, 35th, 65th, and 95th percentiles of caffeine intake distribution. To determine whether the optimal model is linear or non-linear, we conducted a likelihood ratio test and calculated the p-value for non-linearity. Complete model were adjusted for sex (female or male), age (continuous), race/ethnicity (non-Hispanic white, non-Hispanic black, Mexican American, and other races), BMI (<25.0, 25.0-29.9, ≥30), education level (less than high school, high school or equivalent, university or higher), family income-to-poverty ratio (≤1.3, 1.3-3.5, >3.5), smoking status (never smoked, former smoker, current smoker), alcohol consumption (non-drinker, moderate drinker, heavy drinker), hypertension (yes or no), eGFR (continuous), HbA1c (<7%, ≥7%), HDL-C (continuous), TC (continuous), SUA (continuous), HEI-2015 (continuous), red meat intake (oz. eq./d, continuous), white meat intake (oz. eq./d, continuous), total protein (continuous), carbohydrate intake (continuous), total sugars (continuous), total energy (continuous), total saturated fatty acids (continuous), total polyunsaturated fatty acids (continuous), dietary fiber (continuous), vitamin E (continuous), vitamin A (continuous). The pale pink shaded area represents a 95% confidence interval.

*A:* Dose-response relationship between caffeine intake and all-cause mortality in prediabetic patients in NHANES 2003-2018. *P* for non linear < 0.001.

*B:* Dose-response relationship between caffeine intake and cardiovascular mortality in prediabetic patients in NHANES 2003-2018. *P* for non linear =0.0058.

**Table S9. Multivariate adjustment analysis of caffeine intake associated with all-cause mortality and cardiovascular mortality in participants including LDL-C, TG, and fasting glucose in NHANES 2003-2018.**

| **Categories** | **Quartiles of caffeine intake (mg/d)** | | | | ***P* for trend** |
| --- | --- | --- | --- | --- | --- |
|  | **Quartile 1 ≤29.0** | **Quartile 2 29.1-98.0** | **Quartile 3 98.1-200.0** | **Quartile 4 >200.0** |  |
| **All-cause mortality** |  |  |  |  |  |
| No. deaths/total | 879/4772 | 748/4723 | 746/4699 | 793/4720 |  |
| Crude | 1 | 0.76(0.67-0.86) | 0.75(0.65-0.87) | 0.75(0.65-0.86) | 0.004 |
| Model1 | 1 | 0.87(0.75-1.02) | 0.82(0.71-0.95) | 0.81(0.69-0.95) | 0.022 |
| Model2 | 1 | 0.90(0.76-1.07) | 0.81(0.69-0.94) | 0.77(0.65-0.92) | 0.009 |
| Model3 | 1 | 0.88(0.74-1.04) | 0.79(0.68-0.92) | 0.76(0.64-0.91) | 0.011 |
| **CVD mortality** |  |  |  |  |  |
| No. deaths/total | 290/4772 | 260/4723 | 240/4699 | 241/4720 |  |
| Crude | 1 | 0.80(0.64-1.00) | 0.78(0.62-0.99) | 0.70(0.56-0.86) | 0.006 |
| Model1 | 1 | 0.93(0.73-1.20) | 0.90(0.71-1.15) | 0.86(0.67-1.11) | 0.259 |
| Model2 | 1 | 1.24(0.91-1.69) | 0.91(0.68-1.22) | 0.94(0.69-1.29) | 0.361 |
| Model3 | 1 | 1.18(0.86-1.60) | 0.89(0.66-1.18) | 0.90(0.66-1.23) | 0.283 |

Model 1: adjusted for sex (female or male), age (continuous), race/ethnicity (non-Hispanic white, non-Hispanic black, Mexican American, and other races), BMI (<25.0, 25.0-29.9, ≥30), education level (less than high school, high school or equivalent, university or higher), family income-to-poverty ratio (≤1.3, 1.3-3.5, >3.5), smoking status (never smoked, former smoker, current smoker), alcohol consumption (non-drinker, moderate drinker, heavy drinker), and hypertension (yes or no).

Model 2: Model 1 + eGFR (continuous), HbA1c (<7%, ≥7%), HDL-C (continuous), TC (continuous), LDL-C (continuous), TG (continuous), fasting glucose (continuous), and SUA (continuous).

Model 3: Model 2 +HEI-2015 (continuou), red meat intake (oz. eq./d, continuous), white meat intake (oz. eq./d, continuous), total protein (continuous), carbohydrate intake (continuous), total sugars (continuous), total energy (continuous), total saturated fatty acids (continuous), total polyunsaturated fatty acids (continuous), dietary fiber (continuous), vitamin E (continuous), vitamin A (continuous).

**Table S10.** **Baseline characteristics of the lowest and extreme quartiles of caffeine intake after propensity score matching**

| **Characteristic** | **Caffeine intake(mg/d)** | | | | ***P*** | **ASMD** |
| --- | --- | --- | --- | --- | --- | --- |
|  | **Total** | **Quartile 1 ≤29.0** | | **Quartile 4 >200.0** |  |  |
| Patients, n | 5018 | 2509 | | 2509 |  |  |
| Age, years | 56.4 ± 15.4 | 56.6 ± 15.9 | | 56.3 ± 15.0 | 0.4 | 0.022 |
| Sex,n(%) |  |  | |  | 0.8 | 0.01 |
| Male | 2,766 (52.3%) | 1,377 (51.5%) | | 1,389 (53.0%) |  |  |
| Female | 2,252 (47.7%) | 1,132 (48.5%) | | 1,120 (47.0%) |  |  |
| Race/ethnicity,n(%) |  |  | |  | 0.6 | 0.041 |
| Non-Hispanic White | 2,345 (69.1%) | 1,148 (68.3%) | | 1,197 (69.8%) |  |  |
| Non-Hispanic Black | 932 (10.0%) | 475 (10.8%) | | 457 (9.1%) |  |  |
| Mexican American | 790 (7.8%) | 398 (8.2%) | | 392 (7.4%) |  |  |
| Other | 951 (13.1%) | 488 (12.6%) | | 463 (13.6%) |  |  |
| Education level ,n(%) |  |  | |  | 0.7 | 0.031 |
| Less than high | 1,348 (18.1%) | 657 (17.6%) | | 691 (18.5%) |  |  |
| High school grad or equivalent | 1,185 (24.0%) | 601 (24.0%) | | 584 (24.1%) |  |  |
| College or above | 2,485 (57.9%) | 1,251 (58.4%) | | 1,234 (57.4%) |  |  |
| Family income-poverty ratio ,n(%) | | |  |  | >0.9 | 0.016 |
| ≤1.30 | 1,470 (20.3%) | 738 (21.0%) | | 732 (19.6%) |  |  |
| 1.3-3.5 | 2,022 (39.4%) | 1,017 (40.5%) | | 1,005 (38.5%) |  |  |
| >3.5 | 1,526 (40.3%) | 754 (38.5%) | | 772 (41.9%) |  |  |
| BMI, Kg/m2 |  |  | |  | >0.9 | 0.015 |
| < 25.0 | 957 (19.3%) | 471 (18.7%) | | 486 (19.9%) |  |  |
| 25.0-29.9 | 1,644 (32.4%) | 825 (30.6%) | | 819 (34.1%) |  |  |
| ≥30 | 2,417 (48.3%) | 1,213 (50.8%) | | 1,204 (46.0%) |  |  |
| Smoking status ,n(%) |  |  | |  | 0.5 | 0.045 |
| Never | 2,417 (52.2%) | 1,233 (53.8%) | | 1,184 (50.7%) |  |  |
| Former smoker | 1,674 (31.5%) | 831 (30.8%) | | 843 (32.1%) |  |  |
| Current smoker | 927 (16.3%) | 445 (15.4%) | | 482 (17.2%) |  |  |
| Alcohol intake ,n(%) |  |  | |  | 0.8 | 0.026 |
| Never | 3,724 (72.4%) | 1,876 (74.0%) | | 1,848 (70.9%) |  |  |
| Moderate | 731 (15.0%) | 358 (14.3%) | | 373 (15.7%) |  |  |
| Heavy | 563 (12.5%) | 275 (11.7%) | | 288 (13.4%) |  |  |
| Hypertension ,n(%) |  |  | |  | 0.7 | 0.017 |
| No | 2,111 (44.7%) | 1,045 (43.5%) | | 1,066 (45.8%) |  |  |
| Yes | 2,907 (55.3%) | 1,464 (56.5%) | | 1,443 (54.2%) |  |  |
| Diabetes,n(%) | 1,716 (29.2%) | 878 (30.3%) | | 838 (28.1%) | 0.4 | 0.034 |
| Prediabetes,n(%) | 3,302 (70.8%) | 1,631 (69.7%) | | 1,671 (71.9%) | 0.4 | 0.034 |
| Diabetes duration |  |  | |  | 0.8 | 0.026 |
| ≤3 | 3,773 (79.9%) | 1,875 (79.2%) | | 1,898 (80.6%) |  |  |
| 3-10 | 551 (9.4%) | 276 (9.2%) | | 275 (9.6%) |  |  |
| >10 | 694 (10.6%) | 358 (11.5%) | | 336 (9.8%) |  |  |
| HbA_1c_(%) |  |  | |  | >0.9 | 0.002 |
| <7 | 4,280 (88.6%) | 2,141 (88.8%) | | 2,139 (88.4%) |  |  |
| ≥7 | 738 (11.4%) | 368 (11.2%) | | 370 (11.6%) |  |  |
| eGFR(mL/min/1.73m2) | 88.2 ± 21.3 | 87.9 ± 22.6 | | 88.6 ± 20.0 | 0.9 | 0.029 |
| Uric acid(mg/dL) | 5.7 ± 1.4 | 5.7 ± 1.5 | | 5.7 ± 1.3 | >0.9 | 0.002 |
| HDL-Cholesterol (mg/dL) | 51.8 ± 16.0 | 51.4 ± 16.1 | | 52.1 ± 15.9 | 0.2 | 0.006 |
| TC(mg/dL) | 196.3 ± 43.3 | 195.0 ± 43.4 | | 197.6 ± 43.2 | 0.1 | 0.021 |
| HEI-2015 | 51.0 ± 12.2 | 50.9 ± 12.4 | | 51.1 ± 12.0 | 0.7 | 0.028 |
| Total energy(Kcal/d) | 1,887.0 (1,495.5, | 1,849.0 (1,475.4, 2,386.5) | | 1,903.5 (1,524.2, 2,438.5) | 0.2 | 0.016 |
|  | 2,413.5) |  |  |  |  |  |
| Total carbohydrate intake(g/d) | 223.7 (170.5, 285.7) | 222.4 (167.1, 286.1) | | 225.9 (173.2, 284.7) |  |  |
| Total protein(g/d) | 75.5 (58.5, 96.8) | 74.7 (57.3, 95.6) | | 76.1 (59.6, 97.8) | 0.7 | 0.002 |
| Total sugars (g/d) | 90.4 (60.8, 128.0) | 90.6 (61.8, 130.8) | | 90.3 (60.0, 125.2) | 0.2 | 0.004 |
| Dietary fiber(g/d) | 15.7 (11.3, 21.5) | 15.5 (11.0, 21.7) | | 15.8 (11.4, 21.4) | 0.3 | 0.016 |
| Total saturated fatty acids (g/d) | 23.5 (16.5, 32.2) | 23.3 (16.2, 32.4) | | 23.6 (16.9, 32.0) | 0.6 | 0.002 |
| Total polyunsaturated fatty acids (g/d) | 15.9 (11.1, 22.2) | 15.7 (10.5, 21.8) | | 16.0 (11.5, 22.9) | 0.5 | 0.023 |
| Vitamin E(mg/d) | 7.0 (4.8, 10.1) | 6.9 (4.7, 9.8) | | 7.3 (4.9, 10.3) | 0.1 | 0.026 |
| Vitamin A(mg/d) | 0.6 (0.4-0.8) | 0.6（0.4-0.9） | | 0.6（0.4-0.8） | 0.1 | 0.006 |
| Red meat intake(oz. eq./d) | 1.1 (0.0, 2.5) | 1.1 (0.0, 2.6) | | 1.1 (0.0, 2.4) | 0.4 | 0.017 |
| White meat intake(oz. eq./d) | 0.9 (0.0, 2.3) | 0.7 (0.0, 2.2) | | 0.9 (0.0, 2.4) | >0.9 | 0.015 |

Continuous variables with a normal distribution are described as the mean ± standard deviation (SD), while continuous variables with a non-normal distribution are described as the median (interquartile range). Categorical variables are represented by numbers (weighted percentages). BMI: body mass index. HDL-C: HDL cholesterol. TC: total cholesterol. ASMD: absolute standardized mean difference.

**
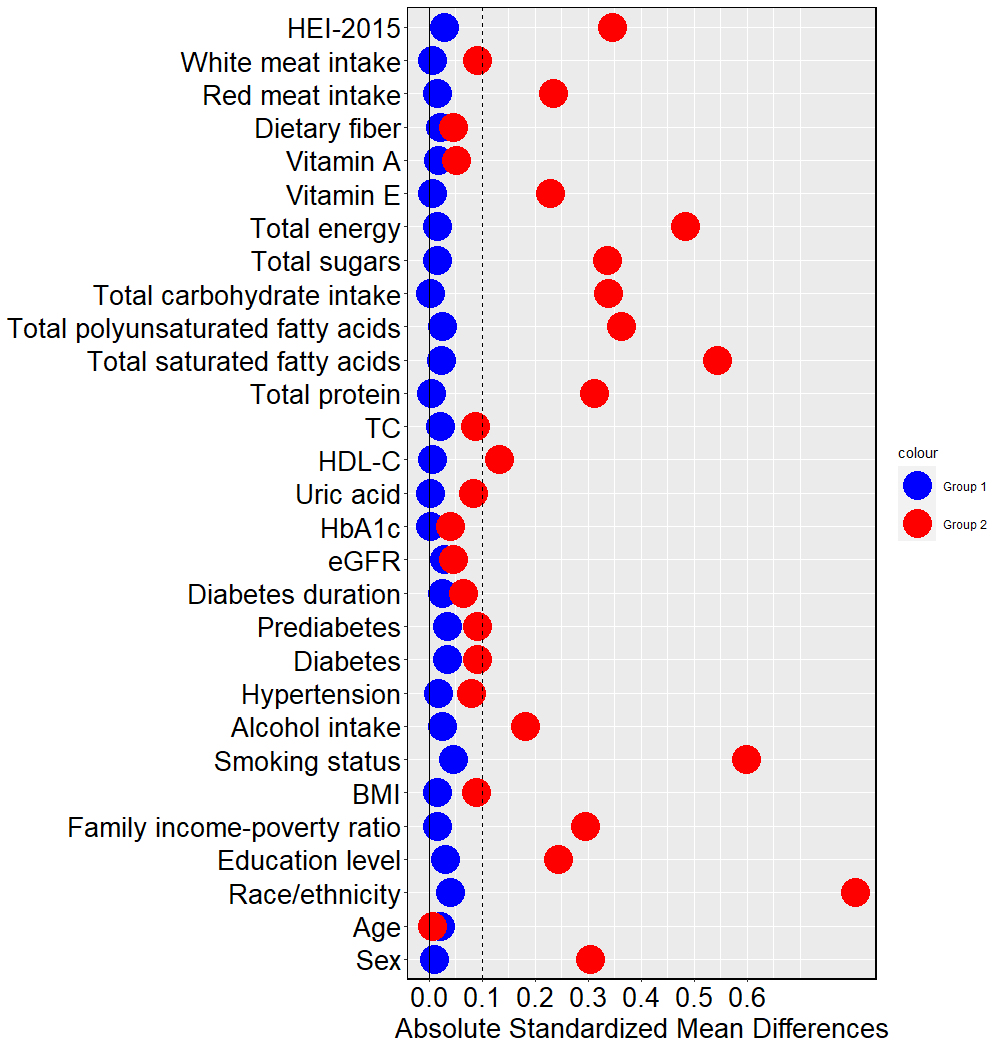
 Figure S5.** **Covariate balance between lowest and extreme quartile individuals before and after propensity score matching.** Red dots indicate absolute standardized mean differences in covariates before propensity score matching. Blue dots indicate absolute standardized mean differences in covariates after propensity score matching. The absolute standardized mean difference of less than 0.1 was considered to indicate a negligible imbalance between the 2 groups (Q1 vs Q4).

**Table S11. Multivariable-adjusted analysis of caffeine intake associated with all-cause mortality and cardiovascular mortality after propensity score matching**

| **Categories** | **Quartiles of caffeine intake (mg/d)** | |
| --- | --- | --- |
|  | **Quartile 1 ≤29.0** | **Quartile 4 >200.0** |
| **All-cause mortality** |  |  |
| No. deaths/total | 497/2509 | 416/2509 |
| Crude | 1 | 0.75(0.65-0.86) |
| Model1 | 1 | 0.78(0.66-0.91) |
| Model2 | 1 | 0.77(0.66-0.9) |
| Model3 | 1 | 0.76(0.65-0.89) |
| **CVD mortality** |  |  |
| No. deaths/total | 152/2509 | 135/2509 |
| Crude | 1 | 0.7(0.56-0.86) |
| Model1 | 1 | 0.86(0.68-1.1) |
| Model2 | 1 | 0.85(0.67-1.08) |
| Model3 | 1 | 0.84(0.66-1.07) |

Propensity score matching was calculated using logistic regression analysis, using a 0.1 standard deviation caliper value of propensity score logit as the threshold.

Model 1: adjusted for sex (female or male), age (continuous), race/ethnicity (non-Hispanic white, non-Hispanic black, Mexican American, and other races), BMI (<25.0, 25.0-29.9, ≥30), education level (less than high school, high school or equivalent, university or higher), family income-to-poverty ratio (≤1.3, 1.3-3.5, >3.5), smoking status (never smoked, former smoker, current smoker), alcohol consumption (non-drinker, moderate drinker, heavy drinker), and hypertension (yes or no).

Model 2: Model 1 + eGFR (continuous), HbA1c (<7%, ≥7%), HDL-C (continuous), TC (continuous), and SUA (continuous).

Model 3: Model 2 +HEI-2015 (continuou), red meat intake (oz. eq./d, continuous), white meat intake (oz. eq./d, continuous), total protein (continuous), carbohydrate intake (continuous), total sugars (continuous), total energy (continuous), total saturated fatty acids (continuous), total polyunsaturated fatty acids (continuous), dietary fiber (continuous), vitamin E (continuous), vitamin A (continuous).

**Table S12.** **Multivariable-adjusted analysis of coffee consumption associated with all-cause mortality and cardiovascular mortality in patients with diabetes and prediabetes in NHANES 2003-2018**

| **Categories** | **Quartiles of caffeine intake (mg/d)** | | | | |
| --- | --- | --- | --- | --- | --- |
|  | **Never** | **0-1 Cup/d** | **1-2 Cups/d** | **2-3 Cups/d** | **≥3 Cups/d** |
| **All-cause mortality** |  |  |  |  |  |
| No. deaths/total | 235/1421 | 1408/8159 | 726/4601 | 405/2392 | 392/2341 |
| Crude | 1 | 0.97(0.79-1.21) | 0.83(0.65-1.05) | 0.85(0.66-1.09) | 0.83(0.64-1.07) |
| Model1 | 1 | 0.80(0.66-0.98) | 0.70(0.56-0.88) | 0.68(0.53-0.87) | 0.71(0.55-0.92) |
| Model2 | 1 | 0.81(0.66-0.98) | 0.70(0.56-0.87) | 0.68(0.53-0.87) | 0.72(0.56-0.93) |
| Model3 | 1 | 0.79(0.66-0.96) | 0.68(0.55-0.84) | 0.67(0.53-0.85) | 0.71(0.55-0.92) |
| **CVD mortality** |  |  |  |  |  |
| No. deaths/total | 74/1421 | 485/8159 | 230/4601 | 125/2392 | 117/2341 |
| Crude | 1 | 1.05(0.76-1.46) | 0.87(0.60-1.25) | 0.87(0.57-1.32) | 0.74(0.49-1.10) |
| Model1 | 1 | 0.89(0.65-1.22) | 0.79(0.55-1.12) | 0.76(0.51-1.13) | 0.76(0.52-1.12) |
| Model2 | 1 | 0.89(0.65-1.22) | 0.78(0.55-1.11) | 0.76(0.52-1.12) | 0.79(0.54-1.15) |
| Model3 | 1 | 0.88(0.65-1.19) | 0.77(0.54-1.09) | 0.76(0.52-1.12) | 0.76(0.52-1.11) |

Model 1: adjusted for sex (female or male), age (continuous), race/ethnicity (non-Hispanic white, non-Hispanic black, Mexican American, and other races), BMI (<25.0, 25.0-29.9, ≥30), education level (less than high school, high school or equivalent, university or higher), family income-to-poverty ratio (≤1.3, 1.3-3.5, >3.5), smoking status (never smoked, former smoker, current smoker), alcohol consumption (non-drinker, moderate drinker, heavy drinker), and hypertension (yes or no).

Model 2: Model 1 + eGFR (continuous), HbA1c (<7%, ≥7%), HDL-C (continuous), TC (continuous), and SUA (continuous).

Model 3: Model 2 +HEI-2015 (continuou), red meat intake (oz. eq./d, continuous), white meat intake (oz. eq./d, continuous), total protein (continuous), carbohydrate intake (continuous), total sugars (continuous), total energy (continuous), total saturated fatty acids (continuous), total polyunsaturated fatty acids (continuous), dietary fiber (continuous), vitamin E (continuous), vitamin A (continuous).

Convert daily caffeine intake to daily coffee consumption (100mg is considered 1 cup)
